# Influence of Copper on *Oleidesulfovibrio alaskensis* G20 Biofilm Formation

**DOI:** 10.3390/microorganisms12091747

**Published:** 2024-08-23

**Authors:** Payal Thakur, Vinoj Gopalakrishnan, Priya Saxena, Mahadevan Subramaniam, Kian Mau Goh, Brent Peyton, Matthew Fields, Rajesh Kumar Sani

**Affiliations:** 1 Department of Chemical and Biological Engineering, South Dakota School of Mines and Technology, Rapid City, SD 57701, USA; payal.thakur@mines.sdsmt.edu (P.T.); vinojvino@gmail.com (V.G.); priya.saxena@mines.sdsmt.edu (P.S.); 2 2-Dimensional Materials for Biofilm Engineering, Science and Technology, South Dakota School of Mines and Technology, Rapid City, SD 57701, USA; 3 Data Driven Material Discovery Center for Bioengineering Innovation, South Dakota School of Mines and Technology, Rapid City, SD 57701, USA; 4 Department of Computer Science, University of Nebraska Omaha, Omaha, NE 68182, USA; msubramaniam@unomaha.edu; 5 Faculty of Science, Universiti Teknologi Malaysia, Johor Bahru 81310, Malaysia; gohkianmau@utm.my; 6 Department of Chemical and Biological Engineering, Montana State University, Bozeman, MT 59717, USA; bpeyton@montana.edu (B.P.); matthew.fields@montana.edu (M.F.); 7 BuG ReMeDEE Consortium, South Dakota School of Mines and Technology, Rapid City, SD 57701, USA; 8 Composite and Nanocomposite Advanced Manufacturing Centre—Biomaterials, Rapid City, SD 57701, USA

**Keywords:** sulfate-reducing bacteria, crystal violet, metal ion, gene expression

## Abstract

Copper is known to have toxic effects on bacterial growth. This study aimed to determine the influence of copper ions on *Oleidesulfovibrio alaskensis* G20 biofilm formation in a lactate-C medium supplemented with variable copper ion concentrations. OA G20, when grown in media supplemented with high copper ion concentrations of 5, 15, and 30 µM, exhibited inhibited growth in its planktonic state. Conversely, under similar copper concentrations, OA G20 demonstrated enhanced biofilm formation on glass coupons. Microscopic studies revealed that biofilms exposed to copper stress demonstrated a change in cellular morphology and more accumulation of carbohydrates and proteins than controls. Consistent with these findings, sulfur (*dsrA*, *dsrB*, *sat*, *aprA*) and electron transport (*NiFeSe*, *NiFe*, *ldh*, *cyt*3) genes, polysaccharide synthesis (*poI*), and genes involved in stress response (*sodB*) were significantly upregulated in copper-induced biofilms, while genes (*ftsZ*, *ftsA*, *ftsQ*) related to cellular division were negatively regulated compared to controls. These results indicate that the presence of copper ions triggers alterations in cellular morphology and gene expression levels in OA G20, impacting cell attachment and EPS production. This adaptation, characterized by increased biofilm formation, represents a crucial strategy employed by OA G20 to resist metal ion stress.

## 1. Introduction

*Oleidesulfovibrio alaskensis* G20 (OA G20) is a sulfate-reducing, Gram-negative, vibrio-shaped bacterium capable of enzymatically reducing sulfate to hydrogen sulfide (H_2_S), enabling the bacteria to respire in anoxic environments. Moreover, OA G20 can form a biofilm, which provides protection against chemical treatments and aids bacterial survival under extreme conditions [1,2]. The formation of a biofilm by sulfate-reducing bacteria (SRB) is a complex process and requires the differential regulation of various genes. Prior studies of biofilm and planktonic cells on several microorganisms have observed altered gene expression patterns [3,4,5]. In the planktonic phase, the bacteria interact with metal to form metal sulfides, while biofilm cells adsorb metal ions through extracellular polymeric substances (EPS). EPS is characterized by the presence of polysaccharides, lipids, and proteins, and it is crucial for both cell adhesion and biofilm formation. Predominantly, OA G20 biofilms are characterized by the presence of large amounts of proteins with minimal polysaccharide content [6,7,8,9,10]. SRBs are of major research interest due to their involvement in influencing microbial-induced corrosion (MIC) either by producing biogenic sulfide or utilizing electron transfer (ET) mechanisms [11,12]. However, SRB can contribute to bioremediation by reducing heavy metals like uranium, copper, chromium, and cadmium, leading to metal precipitation and decreased solubility [13,14,15].

Bacteria utilize copper (Cu) as a co-factor for the activity of various redox enzymes. However, it is toxic at elevated levels. The inhibitory effects of Cu on microbial growth can be due to the production of reactive oxygen species (ROS) or Fe^2+^ substitution in the iron-sulfur cluster of essential enzymes [16]. Therefore, bacteria have adopted a Cu-homeostasis system employing multiple strategies to survive under stress and maintain Cu within safe limits, including passive intracellular and extracellular sequestration and enzymatic detoxification [17,18]. Although considerable research has been conducted on the effects of Cu stress on SRB [19,20,21], the specific mechanisms by which OA G20 produces biofilms under Cu stress remain largely unclear. Thus, it is important to study the gene expression patterns of SRB and analyze the composition and structure of Cu-induced biofilms.

In a previous study, we analyzed the roles of energy metabolism, chemotaxis, and transport systems when OA G20 was exposed to Cu ion stress. These systems are essential for Cu efflux and defending the bacteria against Cu-induced cellular toxicity [22]. However, the role of biofilm formation at high concentrations of dissolved Cu has not yet been investigated in OA G20. This research aimed to examine the influence of dissolved Cu on OA G20 biofilm formation while considering the possible toxic effects of Cu ions on planktonic cells. Our results revealed that under the tested experimental conditions, the biofilms of OA G20 were more resistant to Cu than the planktonic phase. We chose to test genes that have been previously identified as providers of metal resistance to bacteria. These genes counteract oxidative stress or reduce heavy metals via hydrogenase or/and cytochrome [23,24], indirectly through hydrogen sulfide production [25], or via the formation of a biofilm [26].

## 2. Materials and Methods

### 2.1. Bacterial Strain, Culture, and Growth

OA G20 were grown as previously described [22]. A defined medium, lactate-C (anoxic) (pH 7.2), was used as a base medium for the current experimental conditions; the medium’s composition is present in Supplementary Table S1. Now, 100 mL of this prepared lactate-C medium was poured into 125 mL serum bottles and sterilized by autoclaving for 15 min at 121 °C. The medium was made anoxic by sparging ultrapure filter-sterilized nitrogen at 10 psi for 20 min [20,27]. The OA G20 seed cultures were started by inoculating 2 mL of a frozen stock (40% *v*/*v* glycerol stock solution) into each serum bottle described above. The inoculated starter culture was then incubated at 30 °C at 125 rpm for a total of three days. The active starter culture was further sparged for one hour with filter-sterilized ultrapure nitrogen in a chemical exhaust hood to remove any existing hydrogen sulfide (H_2_S). After the removal of the (H_2_S) the cells were centrifuged at 10,000× *g* for 10 min. The supernatant was discarded, and the cell pellets were rinsed with 50 mM of anaerobic phosphate-buffered saline (PBS, pH 7.2). This step was repeated twice, and the washed cells were resuspended in anoxic PBS. A 5% inoculum was used for all of the experiments [20].

### 2.2. Experimental Setup for Planktonic Cells

The effect of Cu on OA G20 was tested in three different setups: (a) serum bottles (planktonic phase), (b) 96-well plates (Biofilm phase, refer to Section 2.3), and (c) glass coupons (GCs) (Biofilm phase, refer to Section 2.4).

A stock solution of CuCl_2_ at a concentration of 0.05 M was prepared and sterilized through a 0.22 μm filter. Different volumes of this solution were added to serum bottles containing 100 mL of anoxic lactate-C medium to achieve final concentrations of 5, 15, 30, 60, 80, and 100 µM. The serum bottles without the addition of CuCl_2_ were treated as the controls. The influence of Cu on OA G20 planktonic cells was investigated for a total of seven days. The planktonic cells were allowed to grow in serum bottles containing variable Cu concentrations at 125 rpm and 30 °C. Total cell protein and OD600 nm were measured for planktonic cells throughout the growth experiment.

### 2.3. Biofilm Formation in 96-Well Plates

To assess the influence of Cu ions on biofilm formation, some preliminary experiments were performed using 96-well plates as previously described [6,28]. To initiate biofilm growth, 200 µL of the bacterial suspension (OD600 nm = 0.04) in the media containing variable Cu concentrations was introduced into the wells of a sterile flat bottom 96-well microtiter plate (Fisher Scientific, Hampton, NH, USA). The plates were then incubated at 30 ℃ to allow biofilm formation, with all the steps performed inside an anaerobic chamber (COY Lab Products, Grass Lake, MI, USA) to ensure anoxic experimental conditions. Four individual 96-well plates were prepared simultaneously, and each plate was monitored on days 1, 3, 5, and 7 to examine biofilm formation. The researchers used the crystal violet (CV) staining method to observe cell attachment. The 96-well plates were removed from the anaerobic chamber and planktonic cells and the spent medium were discarded. The plates were rinsed three times with 50 mM anaerobic PBS (pH 7.2). The wells were stained with 0.1% (*w*/*v*) crystal violet stain for 20 min at room temperature and then rinsed three times with distilled water to remove unbound dye. The bound CV was resuspended in an ethanol: acetone solution (80:20), and then the destained solution was measured at OD595 nm.

### 2.4. Biofilm Growth on Glass Coupons

The GCs [(Borosilicate Glass Disc Coupon from Biosurface Technologies Corporations, Bozeman, MT, USA); (diameter: 12.7 mm; thickness: 3.8 mm)] were sterilized following the US Environmental Protection Agency’s proposed procedure #EPA MLB SOP MB-19 [29]. The GC were sonicated for 5 min in a 1:100 dilution of detergent and tap water. The GCs were then rinsed with reagent-grade water and sonicated for approximately 1 min in reagent-grade water. This process was repeated until all soap residue was removed entirely from the coupons. The clean coupons were then placed in a petri dish and exposed to UV for 15 min before use. Biofilm formation was allowed on GCs submerged in 5 mL of lactate-C media in a 12-well plate (Fisher Scientific, Hampton, NH, USA), inside the anaerobic chamber (COY Lab Products, Grass Lake, MI, USA), under static conditions. On days 1, 3, 5, and 7, the liquid cultures were removed, and the GCs were rinsed with anaerobic 50 mM PBS (pH 7.2). The GCs were then submerged in 3 mL of 0.1% CV (*w*/*v*), incubated at room temperature for 20 min, and then rinsed three times with distilled water to remove unbound dye. Finally, the bound CV was resuspended in an ethanol: acetone solution (80:20). The destained solution was measured at OD595 nm, while the original planktonic cell suspension was measured at OD600 nm. The ratio of OD595 nm to OD600 nm was calculated to determine the relative amount of biofilm formation [6]. To ensure statistical validation, all experiments were conducted in triplicate.

### 2.5. Estimation of Protein and Carbohydrate Content

Samples for the analysis of total protein and carbohydrates were collected as previously described in [4,6]. For growth estimations of planktonic cell samples, cells were collected by centrifugation at 9300× *g* for 10 min at 4 °C and the resulting pellets were resuspended in a solution of 1 mL of 0.7% NaCl (*w*/*v*). For biofilm samples, the samples were collected, as explained earlier [6,30], in which the OA G20 biofilm formed on each GC was carefully rinsed with anaerobic 50 mM PBS (pH 7.2) inside an anaerobic chamber. Then, the GCs were scraped with a single-use sterile cell scraper to collect the biofilm cells. The scraped biomass was then resuspended in 1 mL of 1.5 M NaCl solution and centrifuged at 5000× *g* for 10 min at 25 ℃. The cell-free supernatant was collected as EPS fractions in 1.5mL sterile centrifuge tubes and was subjected to quantitative protein and carbohydrate analysis. The carbohydrate content was determined using a modified phenol-sulfuric method with glucose standards [31]. The protein concentrations were measured using a Pierce^TM^ 660 nm Protein Assay kit (Thermo Fisher Scientific, Waltham, MA, USA) with BSA standards; the samples and the standards were prepared following the instructions provided with the Protein Broad Range (BR) Assay Kits (Thermo Fisher Scientific, Waltham, MA, USA).

### 2.6. RNA Isolation and cDNA Synthesis

On the fifth day of the growth experiment, biofilm samples were collected as previously described [6] to examine their gene expression characteristics. For total RNA extraction, four GCs were used for each experimental condition. First, to remove any planktonic growth, the GCs were carefully rinsed with anoxic PBS (pH 7.2), and the attached biofilm cells were scraped into 1 mL of 50 mM PBS (pH 7.2). The cell pellets were further collected by centrifugation at 10,000× *g* for 10 min at 4 °C, then washed three times with anoxic 50 mM PBS (pH 7.2). The cell pellets were transferred to 2 mL RNase-free microcentrifuge tubes and kept at −80 °C until RNA extraction. The total RNA was extracted using the MasterPure™ Complete RNA Purification Kit protocol from Lucigen (Radnor, PA, USA), following the manufacturer’s instructions. The RNA concentration was then measured using an Qubit RNA assay kit combined with a Qubit 3.0 fluorometer (Thermo Fisher Scientific, Waltham, MA, USA). Also, RNA integrity was analyzed using a Bioanalyzer 2100 system (Agilent Technologies, Santa Clara, CA, USA), with all of the samples consisting of RIN value > 7. To synthesize cDNA, 1 µg of the purified RNA with random hexamers was converted directly into cDNA according to the manufacturer’s instructions for the QuantiTect Rev. Transcription Kit (Qiagen, Hilden, Germany).

### 2.7. RT-qPCR Gene Expression

For this study, the following genes were selected for RT-qPCR: sulfur metabolism, electron transport, cell division, flagellar and polysaccharide synthesis, signal transduction, and stress response (Supplementary Table S2). The reactions were conducted in an ABI 7500 instrument from Applied Biosystems, Waltham, MA, USA, using version 2.0.6 software and the PowerSYBR Green Master Mix (Thermo Fisher Scientific, Waltham, MA, USA) following the manufacturer’s guidelines. The analyses were conducted automatically, using the default settings, which included the threshold and quantification cycle (Cq) values. The experiments were performed in 96-well plates, with a reaction mixture consisting of 2 µL of cDNA, 10 µL of SYBR Green Master Mix, 0.5 µL of 100 µM of each primer, and 7 µL of RNase-treated water. The reaction was initiated with an initial denaturation step at 95 °C for 5 min, followed by 40 amplification cycles of 95 °C for 20 s, 60 °C for 20 s, and 72 °C for 20 s. To analyze the PCR results, relative quantification was performed using the *recA* (housekeeping) gene as a reference gene. The 2^-∆∆CT^ method [32] was used to evaluate the relative expression ratio between each planktonic and biofilm sample.

### 2.8. Scanning Electron Microscopy (SEM)

On day 5 of growth, biofilm samples were collected and prepared for SEM analysis as previously described [22]. The GCs were washed with anoxic 50 mM PBS (pH 7.2) and placed in a fixing solution containing 2% glutaraldehyde (*w*/*v*) and 0.1 M sodium cacodylate buffer (pH 7.2). The biofilms were then fixed overnight, washed with distilled water four times for 20 min each, and dehydrated with increasing concentrations of ethanol. Later, the samples were dried in a desiccator until all the ethanol had evaporated. Next, a Helios 5 CX FIB-SEM (Thermo Fisher Scientific, Waltham, MA, USA) was used to observe biofilm formation on GC under variable Cu concentrations.

### 2.9. Confocal Microscopy

Biofilm samples were also analyzed for cell viability and the presence of exopolysaccharides (EXP) using fluorescent stains and a spinning disc confocal microscope (Nikon, Nishio, Japan) using an 60× water immersion objective. The samples were prepared by optimizing protocols as previously explained [33,34,35]. The biofilm samples were washed with anoxic 50 mM PBS (pH 7.2), and the GCs were stained with SYTO 9 (3.34 mM in DMSO) (Invitrogen, Waltham, MA, USA) according to the manufacturer’s instructions. Briefly, 200 µL of SYTO 9 solution was gently applied onto each coupon’s surface, and the samples were incubated in the dark for 15 min at room temperature. The GCs were washed twice with 50 mM PBS (pH 7.2) to remove excess stain. To identify the presence of EXP, the GCs were stained using Calcofluor White solution (1:1 mixture of Calcofluor White and 10% *w*/*v* potassium hydroxide). Then, 15 μL of the Calcofluor White mixture was applied to the surface of the coupon. The GCs were placed in the dark at room temperature for 1 min and then washed with 50 mM PBS (pH 7.2) before capturing the images.

It is important to note that a maximum concentration of 30 µM was selected to investigate the effect of Cu on the gene expression analysis of OA G20 biofilms. We selected 30 µM as the highest Cu concentration to test because RNA yields were insufficient at higher Cu levels. While higher Cu concentrations of 60, 80, and 100 µM were used to analyze biofilm formation, these experiments were limited to growth measurement (OD600 nm), protein quantification (OD660 nm), CV assay, and SEM analysis.

## 3. Results and Discussions

### 3.1. Growth of OA G20 Under Variable Cu Concentrations

**Figure 1:**
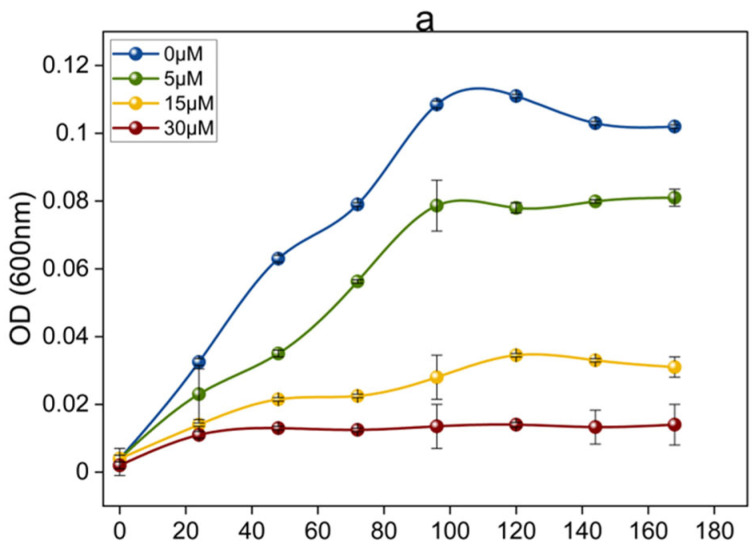

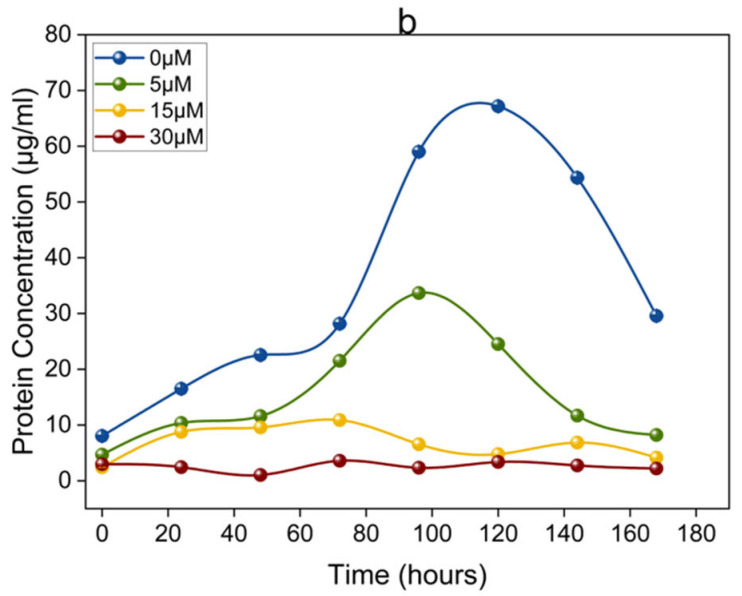
(**a**) Growth curve of planktonic OA G20 in lactate-C medium supplemented with 0, 5, 15, and 30 µM Cu in serum bottles; (**b**) Influence of variable Cu concentrations of 5, 15, and 30 µM on planktonic growth of OA G20 as a function of total protein (OD 660 nm) grown in serum bottles containing lactate-C medium. Each data point indicates the average values of three biological triplicates and the error bars represent ± standard deviations of the means. Error bars smaller than the data point symbols are obscured by data legends.

The current experiment investigated the influence of Cu on the growth of OA G20 planktonic and biofilm cells. A uniform decrease in the planktonic growth with an increase in Cu ion concentrations was observed (Figure 1a). At 5, 15, and 30 μM Cu ion concentrations, the maximum specific growth rate (μ_max_) of planktonic cells was approximately 0.012 h^−1^, 0.011 h^−1^, and 0.010 h^−1^, respectively, compared to 0.015 h^−1^ in control (0 µM CuCl_2_). Additionally, for planktonic cells, the total cellular protein was measured to eliminate obstructions in OD600 nm measurements due to the presence of any Cu sulfide precipitates produced during the growth experiments (Figure 1b). Also, measurements of the total cellular protein in planktonic cells showed a similar trend of consistent decrease in protein concentrations by 55.8%, 81.1%, and 92.8% for the 5 μM, 15 μM, and 30 μM Cu, respectively, in comparison to 0 µM. The effect of Cu toxicity on OA G20 planktonic growth is presented in Figure 1a,b. Furthermore, our findings are consistent with the results of previous studies of metal toxicity in SRB [20,22,36]. It is interesting to note that when OA G20 was grown in microtiter plates (refer to Section 3.2.1) with varying Cu ion concentrations, increased cell attachment was observed with the increase in Cu ion concentrations. Due to these differences in the levels of cell attachment, biofilm formation by OA G20 cells was further determined.

### 3.2. Biofilm Formation Under Copper Stress

#### 3.2.1. Biofilm Growth in 96-Well Plates

**Figure 2:**
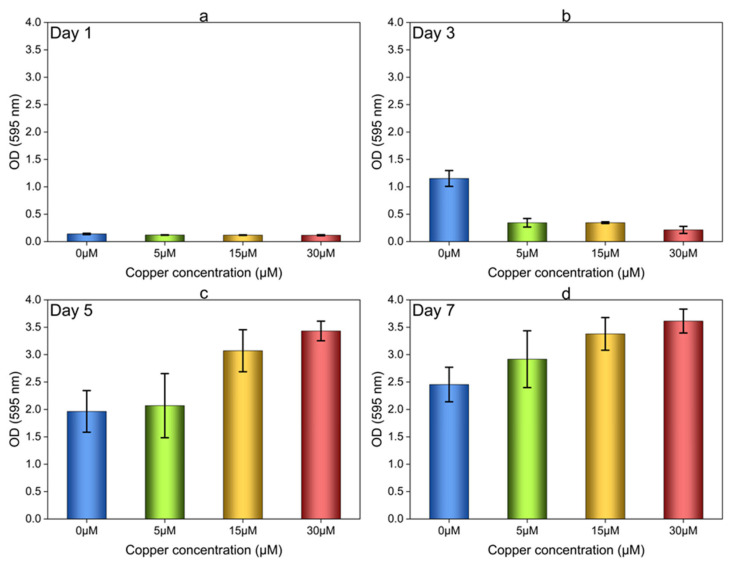
Effect of 5, 15, and 30 µM Cu ions on cell attachment and CV staining for OA G20 biofilms grown in 96-well plates. OA G20 biofilms were grown inside the anaerobic chamber at 30 °C for a total of 7 days in lactate-C medium. The biofilms were stained with 0.1% CV (*w*/*v*) and then bound CV was dissolved in 80:20 ethanol: acetone solution followed by measuring absorbance at 595 nm (A595). (**a**) Represents cell attachment observed on Day 1 of OA G20 growth; (**b**) Absorbance evaluated on Day 3 of biofilm growth; (**c**–**d**) Biofilm quantification on Day 5 and 7 for OA G20. The experiment was performed in biological triplicates and technical triplicates for every sample. The measurements recorded at A595 were averaged, and the error bars indicate ± standard deviations.

Initially, the effect of Cu ions on OA G20 biofilm formation was determined in 96-well plates under static conditions inside an anaerobic chamber. Figure 2 represents the results of OA G20 biofilm formation on microtiter plates on days 1, 3, 5, and 7. The differences in biofilm formation under variable Cu ion concentrations (5 µM, 15 µM, and 30 µM) was quantified by measuring the CV dye on 96-well plates. It can be seen from Figure 2a that no significant cell attachment was observed on day 1. On day 3 of the biofilm growth experiment, cell attachment in the 96-well plates was observed, with more retention of the CV dye in control wells (no added Cu, 0 µM) relative to the samples containing Cu. To further investigate OA G20 biofilm formation, the microtiter plates were incubated for a total of seven days, maintaining anoxic conditions. On days 5 and 7, it was observed that OA G20 cells grown in the media supplemented with Cu ions exhibited a larger variation in the level of cells attached to the microtiter plate. Moreover, higher retention of crystal violet was seen in wells with 5, 15, and 30 µM of Cu, despite their having lower levels of planktonic growth than the controls, indicating a strong influence of Cu ions on the level of biofilm formation. In the wells containing 5 μM of Cu, the amount of CV retained was roughly 1-fold higher than that of the control biofilms on days 5 and 7. Moreover, the wells supplemented with 15 and 30 μM of Cu showed even greater retention of CV stain, with approximately 1.5 to 2-fold higher retention in comparison to the controls. As the preliminary experiments with CV staining showed larger variations in the cell attachment with increasing concentrations, the effect of Cu on biofilm growth and quantification was now tested in 12-well plates containing GCs.

#### 3.2.2. Biofilm Formation on Glass Coupons

**Figure 3:**
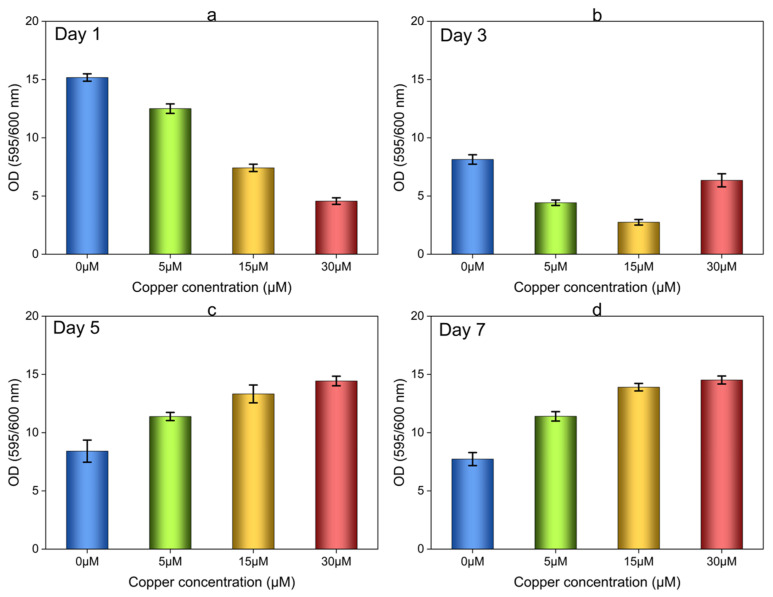
Crystal violet staining for OA G20 biofilms grown on GCs. OA-G2O biofilms were grown in 12-well plates inside the anaerobic chamber at 30℃, static conditions. The biofilms were grown for a total of 7 days in a lactate-C medium supplemented with variable Cu concentrations of 0, 5, 15, and 30 µM. (**a**–**d**) represent spectrophotometric quantification of OA G20 biofilms on days 1,3,5 and 7, respectively. The biofilms were stained with 0.1% CV (*w*/*v*) and then bound CV was dissolved in 80:20 ethanol: acetone solution followed by measuring absorbance at 595 nm. Simultaneously, the planktonic phase of the biofilm samples was also measured for growth at OD 600 nm. The graph values are expressed as a ratio of OD (595/600 nm) adjusted to variations in cell density. The experiment was conducted in biological triplicates.

OA G20 biofilms were grown in 12-well plates as described in Section 2.4. The biofilms were allowed to develop for a total of 7 days, and on every alternate day (i.e., days 1, 3, 5, and 7) the coupons were stained with CV to assess biofilm formation. To account for any variations in total growth (measured at OD600 nm) and biofilm formation (measured at OD595 nm), the relative biofilm data was calculated as the ratio of OD595 nm to OD600 nm for the GCs. This OD 595/600 ratio enabled the normalization of the biofilm formation value to the total cell growth, eliminating differences in overall growth levels between the samples [37]. From Figure 3c,d, it can be observed that on days 5 and 7, the relative biofilm formation OD (595/600) value for the control samples was approximately 2-fold smaller compared to biofilms formed on GCs supplemented with 15 μM and 30 μM Cu. The formation of biofilms on the GCs under high Cu concentrations is consistent with the results described in the section above.

It should be noted that cell attachment occurred on the GCs from day 1, but not in the 96-well plates under the same experimental conditions. This discrepancy can be attributed to the differences in the material properties of the two surfaces. It has been previously suggested that cell attachment and initial biofilm formation can be greatly influenced by a wide range of surface characteristics, including surface area, hydrophobicity, charge, and roughness [38,39,40]. Prior studies have reported that polystyrene surfaces tend to promote more cell attachment compared to glass, which contradicts the findings of our experiments. However, we cannot rule out the possibility that better cell attachment on the glass surface in our study is due to the larger surface area compared to the small area in plates [38]. The observation of higher OD 595/600 values on day 1 (Figure 3a) for all of the variable Cu concentrations, compared to the OD 595/600 values on days 3 (Figure 3b), 5 (Figure 3c), and 7 (Figure 3d), remains unexplained and requires further investigation.

Collectively, this study suggests that biofilm cells are more resistant to Cu in comparison to OA G20 in their planktonic state. Over 7 days, an increase in cell attachment was noticed in both 96-well plates (Supplementary Figure S1) and on GCs (Supplementary Figure S2) when the cells were allowed to grow in media supplemented with varying Cu ion concentrations. In both experimental setups, it was observed that cells grown in media exhibiting higher Cu ion concentrations showed greater cell attachment, indicating the role of Cu in influencing biofilm formation and EPS production to protect the cells from Cu toxicity. As a result of these findings, further investigations were carried out to characterize the EPS of Cu-induced biofilms.

### 3.3. Copper Resistance Level of OA- G20 Planktonic and Biofilm Cells

**Figure 4:**
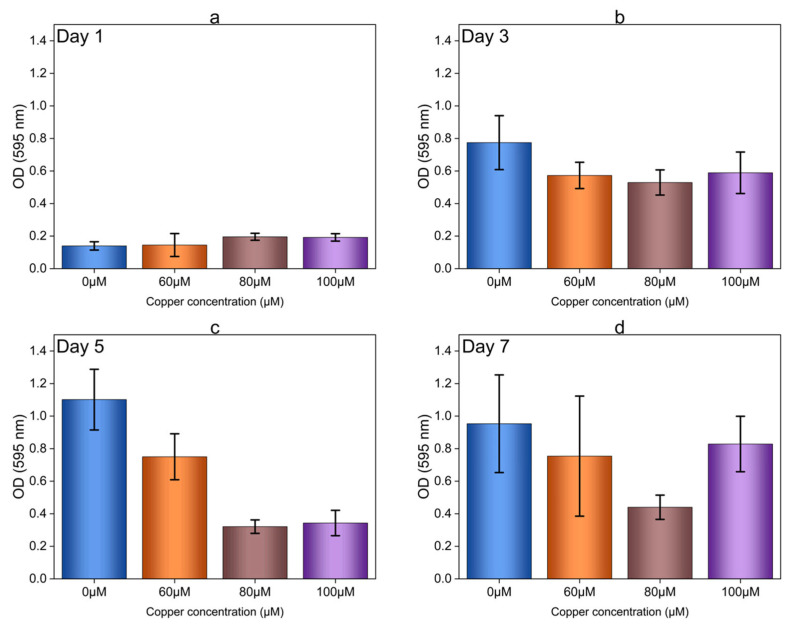
Effect of high Cu ion concentrations (60, 80, and 100 µM) on cell attachment and CV staining for OA G20 biofilms grown in a microtiter plate. OA G20 biofilms were grown inside the anaerobic chamber at 30 °C for a total of 7 days in a lactate-C medium. The biofilms were stained with 0.1% CV (*w*/*v*) and then dissolved in 80:20 ethanol: acetone solution followed by measuring absorbance at 595 nm (A595). (**a**–**d**) represent the formation of Cu-induced biofilms compared to control on microtiter plates on days 1, 3, 5, and 7, respectively. The experiment was performed in biological triplicates and technical triplicates for each sample. The recorded values at (A595) were averaged, and the error bars indicate ± standard deviations.

In our previous study on the toxic effects of Cu on OA G20 [22], we established a baseline indicating altered cell morphology and growth characteristics when the bacteria were exposed to Cu stress. Comparable patterns were observed in our current study, which documented a decrease in the growth rate with increasing Cu ion concentrations. Additionally, planktonic cells were subjected to higher concentrations of Cu; 60, 80, and 100 µM (Supplementary Figure S3). It was observed that the growth of OA G20 planktonic cells was inhibited at concentrations greater than 15 µM of Cu ion concentration (Figure 1 and Figure S3), compared to the controls. However, the biofilms of OA G20 grown on the 96-well plates (Supplementary Figures S1 and S4) and GCs (Figure S2) exhibit greater resistance to Cu than the planktonic cells. Even at Cu concentrations up to 100 µM, complete inhibition of OA G20 biofilm formation was not observed (Supplementary Figure S4). It is worth noting that although the attachment of cells on 96-well plates for days 3,5 and 7 (Figure 4b–d) was observed through CV experiments using higher Cu concentrations (60, 80, and 100 µM), SEM analysis revealed that the biofilms formed under these high Cu concentrations were non-uniform compared to the control (0 µM). Furthermore, the cell attachment (OD595 nm) quantified through CV studies for 100 µM was approximately 3-fold lower on day 5 and 1-fold lower on day 7 compared to the controls (Figure 4c,d).

Our results suggest that planktonic cells of OA G20 were more susceptible to Cu than cells within the biofilm. These findings align with previous research on the Cu and cadmium (Cd) resistance of sulfate-reducing bacteria (SRB) biofilms, in which biofilm formation was evaluated in conditions including up to 200 µM concentrations of metal ions of Cu and Cd [8,9]. It is worth considering that the concentration values in their study were 10 times higher than those observed in our experiments. This difference can be attributed to the fact that they investigated biofilms of SRB consortia, which are generally known to demonstrate higher resistance to metal ions than single cultures, similar to the OA G20 pure cultures used in our current experiment. Also, due to insufficient biomass recovery from biofilms grown in the high Cu concentrations of 60, 80 and 100 µM, further experiments were only conducted with the samples supplemented with the highest Cu concentration of 30 µM. Additionally, the RNA extracted from the 60, 80 and 100 µM biofilm samples was of poor quality and did not yield sufficient quantities to further conduct gene expression analysis.

### 3.4. Total Protein and Carbohydrate Analysis of Copper-Induced Biofilms

**Figure 5:**
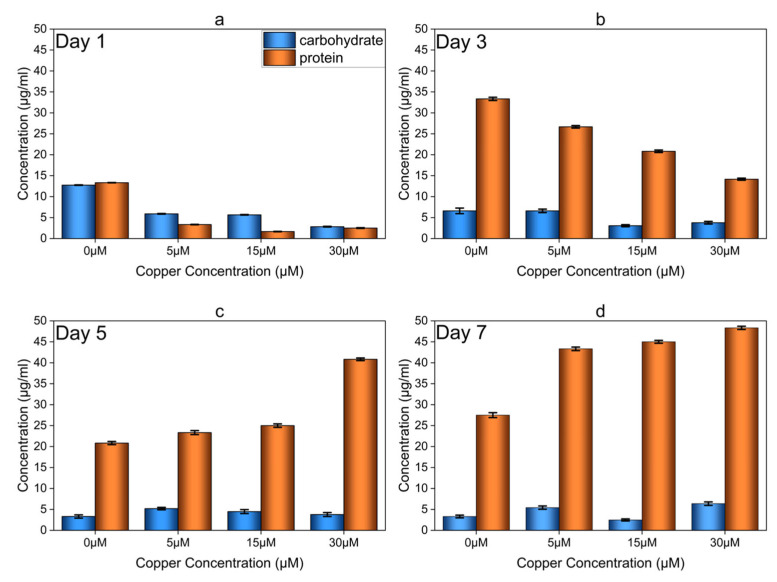
Analysis of OA G20 Cu-induced and control biofilms protein and carbohydrate content. The biofilms were extracted from GCs on days 1, 3, 5 and 7 (**a**–**d**) and tested for the presence of protein and carbohydrate levels. The experiment was performed in biological triplicates.

The total protein and carbohydrate content of the biofilms was quantified on days 1, 3, 5, and 7 (Figure 5) as previously described [6,30] for the growth assessment of OA G20 biofilms and to identify appropriate sampling points for gene expression and microscopic analyses. Biofilms developed in higher Cu concentrations had significantly higher total protein and carbohydrate content compared to the control biofilms (no added Cu). Notably, large amounts of protein were accumulated within the biofilms formed in an environment containing 30 µM Cu. The Cu-induced biofilms (30 µM) showed approximately a 2-fold increase in total protein and carbohydrate accumulation on day 7 compared to the control biofilm (Figure 5d). The increase in protein and carbohydrate content in Cu-induced biofilms suggests their active role in the uptake or retention of Cu ions. The biofilm reached steady-state conditions on day 5 for the control and day 7 for Cu-induced biofilms. The 30 µM-biofilm had a protein content of 48.33 µg/mL and a carbohydrate content of 6.37 µg/mL, resulting in an estimated carbohydrate–protein (C:P) ratio of 0.13 (µg/µg). These results are consistent with previous studies on *Desulfovibrio vulgaris* biofilms [6,41]. These studies revealed that *D. vulgaris* produces a protein-rich biofilm matrix, in which the bacteria are dependent on proteins for matrix stability and biofilm formation.

One of the key survival strategies adopted by bacteria when exposed to toxic metal ions is cell adhesion, thereby promoting biofilm formation [42,43]. Exposure to heavy metals affects cells’ attachment capabilities along with influencing structural changes within the biofilm matrix. In response, the bacteria produce a more compact and resistant biofilm architecture. Previous studies have reported the role of extracellular polymeric matrix in protecting cells from toxic metals to be facilitating their sequestration, immobilization, and precipitation [4,8,44]. Moreover, increasing the amount of carbohydrates or EPS in metal-induced biofilms compared to non-exposed biofilms can enhance the metal entrapment process [4,9,45,46]. Toxic metals can also bind to functional groups present in the EPS, such as carboxyl, hydroxyl, and amino groups. Consequently, the diffusion of metal ions can be restricted by the presence of both positive and negative charges within the EPS, thereby reducing their bioavailability in the surrounding environment and rendering additional metal resistance to the bacteria [42].

### 3.5. Microscopic Analysis of OA G20 Biofilm

**Figure 6:**
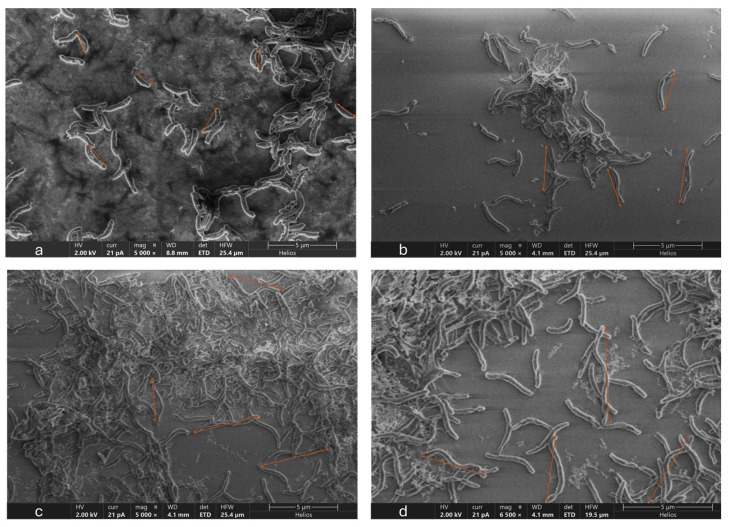
SEM images of OA G20 grown on GCs in lactate-C media (**a**) no-Cu; (**b**) 5 µM; (**c**) 15 µM; (**d**) 30 µM, respectively. OA-G2O biofilms were grown in 12-well plates inside the anaerobic chamber at 30 °C, static conditions. SEM images were taken on day 5 of OA G20 growth on GCs. The images were acquired at a scale bar of 5 µm, specified at the bottom right. The brown arrows on the images represent differences in cell length for cells grown in different Cu concentrations. Shorter arrows indicate normal rod-shaped cells in the control, while longer arrows show elongated cells observed at 30 µM Cu concentration.

**Figure 7:**
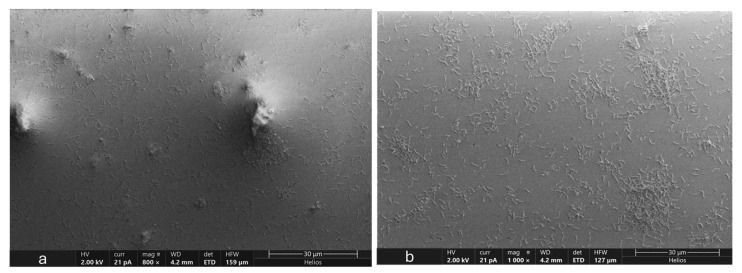

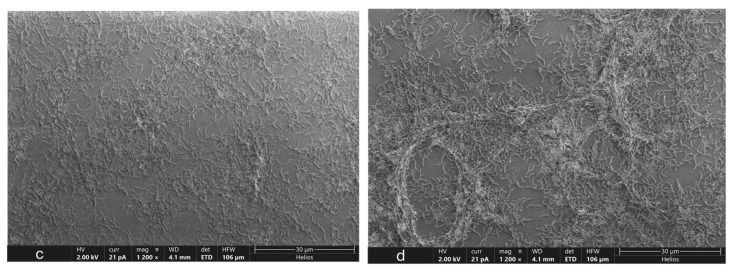
SEM images of OA G20 biofilms grown on GCs in lactate-C media (**a**) no-Cu; (**b**) 5 µM; (**c**) 15 µM; (**d**) 30 µM, respectively. SEM images were taken on day 5 of OA G20 growth on GCs. The images were acquired at a scale bar of 30 µm, specified at the bottom right. (**a**–**d**) represent variations in OA G20 biofilm formation when grown in 0, 5, 15, and 30 µM Cu ion concentrations.

**Figure 8:**
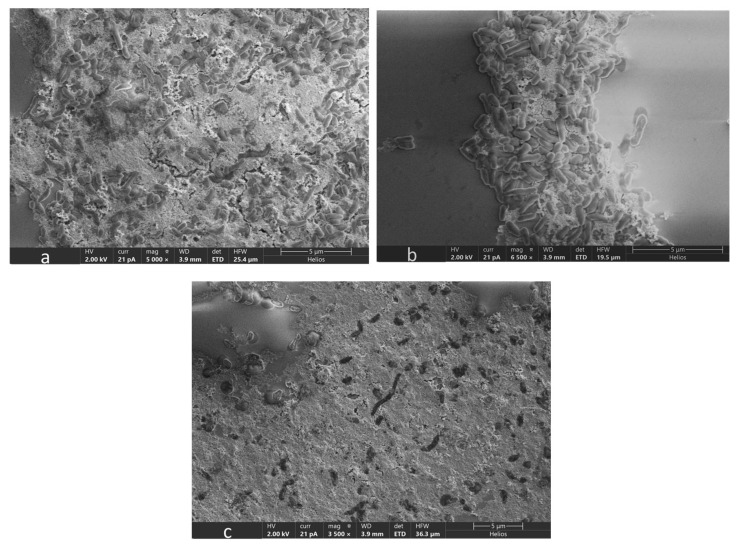
SEM images of OA G20 biofilms grown on GCs in lactate-C media supplemented with high Cu concentrations of (**a**) 60 µM; (**b**) 80 µM; (**c**) 100 µM, respectively. SEM images were taken on day 5 of OA G20 growth on GCs. The images were acquired at a scale bar of 5 µm, specified at the bottom right. (**a**–**c**) represent variations in OA G20 biofilm formation when grown in 60, 80, and 100 µM Cu ion concentrations.

The results of our SEM analysis of OA G20 biofilms grown on GCs with/without Cu are presented in Figure 6 and Figure 7. The biofilm images were obtained at scale bars of 5 µm (Figure 6) and 30 µm (Figure 7). It was observed that dense biofilms were formed on GCs grown in media containing 15 µM and 30 µM Cu ion concentrations relative to the control samples. The biofilm samples exposed to 30 µM and 15 µM Cu ions exhibited elongated cells compared to the controls. The cell elongation observed under Cu stress is in accordance with our earlier findings on OA G20 planktonic cells grown under different Cu concentrations [22]. Interestingly, even at the highest examined Cu ion concentration (100 µM), the biofilm formation was not inhibited under our current tested conditions. It is important to consider that there was a significant change in the structure of biofilms grown in 30 µM compared to 100 µM Cu. Biofilms exposed to high Cu ion concentrations (>30 µM) did not form organized biofilms, and disrupted cell-to-cell interactions were observed with no change in cellular morphology (Figure 8). Our current study did not observe inhibitions of biofilm formation under the tested Cu ion concentrations (≤100 µM), suggesting that higher Cu concentrations might be required to inhibit OA G20 biofilm formation in lactate-C medium.

**Figure 9:**
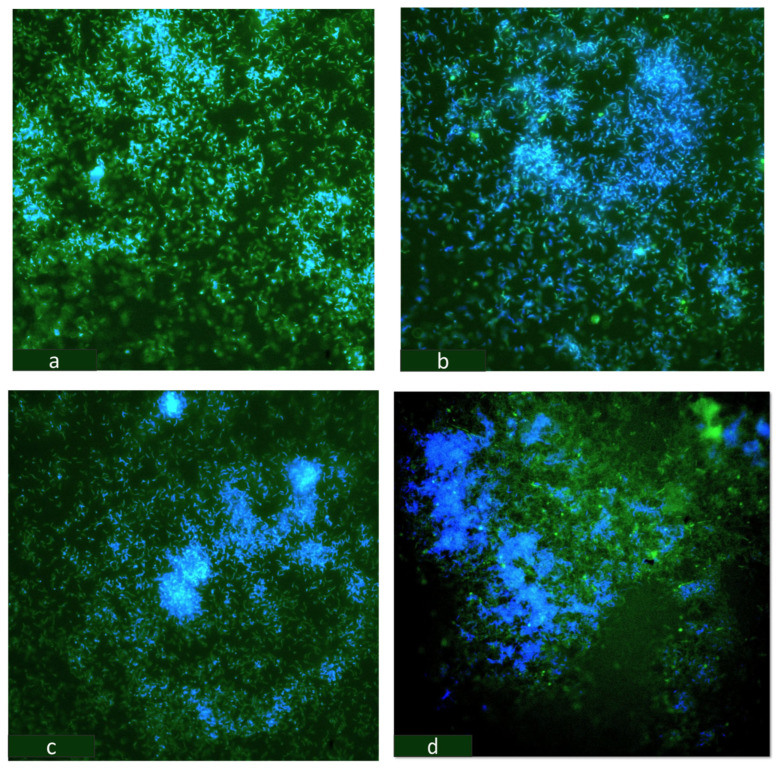
OA G20 biofilm grown on GCs in lactate-C media (**a**) no-Cu (control); (**b**) 5 µM-Cu; (**c**) 15 µM-Cu; (**d**) 30 µM-Cu stained with 200 µL of Syto9 followed by staining the EXP with Calcofluor White stain. The biofilms were analyzed using a confocal microscope. Magnification bar = 10 µm.

CLSM has been employed to examine the structure and composition of biofilms, as well as the viability of cells in various microorganisms [47,48]. In our study, we employed confocal microscopy to investigate the influence of Cu on biofilm formation on GCs (Figure 9). Thick biofilms with densely packed colonies were observed on the coupons supplemented with 30 µM Cu. To further examine the presence of EPS, the coupons were treated with Calcofluor White solution. The results revealed a more intense blue region in the 30 µM (Figure 9d) biofilms than in the controls (Figure 9a). Similar results were observed for *S. aureus* biofilms grown under shear stress. COMSTAT analysis revealed that the sheer stress-induced biofilms retained 5-fold more Calcofluor White stained EPS in comparison to the controls [49].

Changes in cellular morphology, EPS production, and increased biofilm formation under stress have deepened our understanding of the detoxification mechanism utilized by the bacteria to survive under unfavorable conditions [50]. Previous studies on bacteria such as *Pseudomonas putida* have confirmed that bacteria adopt a smaller cell size to detoxify cadmium. In contrast, irregular, giant, and elongated cells were observed in *Pantoea agglomerans* cells exposed to heavy metals like Cu, lead, and chromium [50,51]. Also, Sani et al. reported that elongated cells were not observed when SRB were grown under strict anaerobic conditions, indicating the absence of oxidative stress [20]. Our SEM and confocal results also support these findings that bacteria employ several strategies like cell elongation and more EPS production [52] to survive under stressful conditions.

### 3.6. Relative Expression Analysis of OA G20 Biofilms

**Figure 10:**
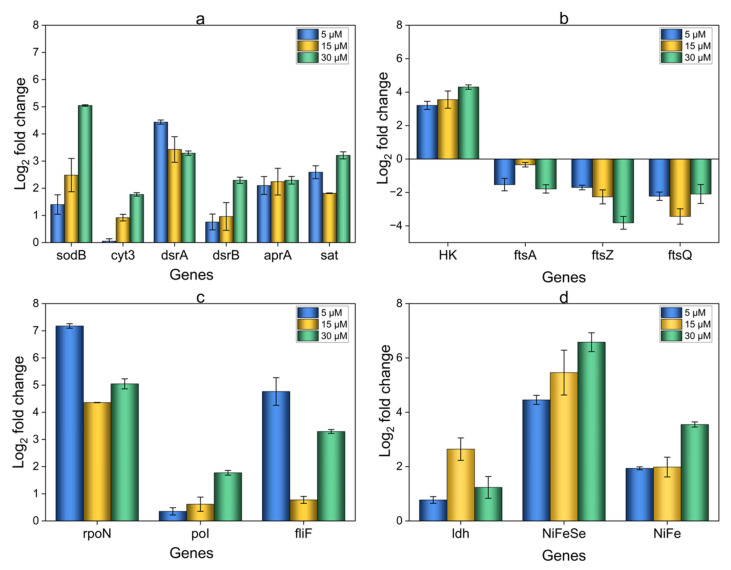
RT-qPCR was performed on cDNA synthesized from 1 µg of total RNA extracted from OA G20 biofilms. The biofilms were allowed to grow for a total of five days on GCs in a lactate-C medium containing varying Cu concentrations of 5, 15, and 30 µM, inside an anaerobic chamber under static conditions. The GCs were scraped using a sterile scraper and RNA was isolated for each sample. The expression level of genes mentioned above is calculated relative to the control (0 µM-Cu). The experiment was performed in biological triplicates. (**a**) genes involved in sulfur metabolism, ET and stress response; (**b**–**c**) expression analysis of genes involved in cell division and two-component, EXP, and flagellar biosynthesis; (**d**) genes involved in ET process.

The possible metabolic processes involved in OA G20 biofilm formation under Cu ion stress include sulfur metabolism, polysaccharide biosynthesis, and electron transport [53]. Differential expression analysis was therefore performed on genes associated with these mechanisms. The study revealed both upregulated and downregulated genes for biofilms grown in 5, 15, and 30 µM Cu concentrations compared to the controls (Figure 10). Most of the genes showed an increase in their expression level in Cu-induced biofilms compared to the controls. The genes *dsrA* (Dde_0526), *dsrB* (Dde_0527), *aprA* (Dde_1109), and *sat* (Dde_2265), play key roles in the dissimilatory sulfate reduction pathway, and they exhibited differential upregulation in their expression levels relative to the controls. The most upregulated gene in the sulfur metabolism (log_2_FC = 4.434) was *dsrA* (Dde_0526), as found in the 5 µM-biofilms. However, no significant difference was observed between the 15 µM (log_2_FC = 3.426) and 30 µM (log_2_FC = 3.292) expression levels. Moreover, the *sat* (Dde_2265) and *aprA* (Dde_1109) genes did not display significant differences in expression levels across the tested Cu concentrations (Figure 10a). The coordinated action of these genes allows OA G20 to convert the sulfate to H_2_S. The *Sat* converts the internalized sulfate to adenosine-5′-phosphosulfate (APS), which is then reduced to sulfite by *aprAB*. Now, the *dsrAB* enzyme complex initiates the reduction of sulfite, partially converting it to H_2_S. The final reduction is carried out by *dsrC*, which facilitates the final reduction of sulfite to H_2_S. The upregulation of *dsrAB*, *aprA*, and *sat* may indicate an adaptive response triggered under Cu stress to meet higher energy demands required for biofilm formation [43,54,55,56]. The use of KEGG pathway map00920 provided a framework for identifying key genes involved in dissimilatory sulfate reduction [57]. Our analysis revealed a lack of clear pattern in the upregulation of the above-mentioned genes (*dsrA*, *dsrB*, *aprA*, and *sat)* in response to increasing Cu ion concentrations, indicating that OA G20 at high Cu concentrations might be saving energy for other essential metabolic functions. This irregularities in the gene expression levels of stress-induced biofilms need further investigation.

The bacterial signal transduction system is characterized by the presence of a histidine kinase (HK) and a response regulator, known as a two-component regulation. The KEGG pathway map02020 provided valuable insights into bacterial signaling mechanisms relevant to our study [58]. In reaction to a signal (including pH, temperature, and ions), HK autophosphorylates and then transfers the phosphate group to the amino-terminal of the response regulator [59,60]. This phosphorylation cascade regulates various developmental processes like sporulation and biofilm formation in bacteria [59]. Interestingly, the gene coding for HK (Dde_3717), which is an integral part of a two-component signal transduction system, was significantly upregulated with log_2_FC = 4.302, log_2_FC = 3.550, and log_2_FC = 3.208 in 30 µM, 15 µM, and 5 µM biofilms, respectively (Figure 10b). Previous studies have indicated that two-component signal transduction systems play a role in biofilm formation, including a type of hybrid kinase involved in signal transduction, an essential component of cell-to-cell communication and differentiation. The increased expression of HK (Dde_3717) in Cu-induced biofilms can be attributed to its dual role in Cu detoxification and biofilm formation. Histidine kinases allow the bacteria to sense and adapt to environmental stimuli. The elevated levels of Cu act as a stress signal that triggers signaling pathways involving histidine kinases. Cu stress influences biofilm-specific responses by creating localized concentration gradients, accumulating Cu ions within the biofilm matrix. The presence of these Cu ions is detected by histidine kinase, regulating the activation of genes involved in biofilm formation and stability. Moreover, HK plays a key role in regulating gene expressions involved in Cu detoxification, transport, and efflux pumps. For example, in *E. coli*, a two-fold increase in histidine kinase (HK) expression was observed under Cu stress. The *cusS* gene encoding histidine kinase detects high levels of silver/Cu in the periplasmic space of *E. coli.* [61,62,63,64]. The role of sigma factor *rpoN* is well-established in the regulation of biofilm formation, nitrogen assimilation, virulence, resistance, and motility [65,66]. Based on the data shown in Figure 10c, *rpoN* (Dde_3097) shows the highest upregulation, with log2FC = 7.176 in 5 µM-biofilms. Similarly, 15 µM and 30 µM biofilms also demonstrate upregulation of *rpoN* compared to controls (0 µM) with log2FC = 4.357 and log2FC = 5.046, respectively. Xiaoxiang Liu et al. explored the role of *rpoN* mutants in *Pseudomonas fluorescens* on biofilm formation and stress resistance. The results showed that *rpoN*-defective mutants did not form robust biofilms on solid and semi-solid agar plates and also showed significant downregulation of genes related to reduced resistance to heat and antibiotic stress [66]. These results suggest that *rpoN* genes positively regulate biofilm formation and stress resistance in bacteria. The *rpoN*-defective mutants studied by other researchers in *Pseudomonas aeruginosa*, *Vibrio mimicus*, and *Yersinia pseudotuberculosis* indicated similar results validating the role of the *rpoN* gene in the regulation of cell adhesion, cell-cell communication, and resistance [67,68,69]. It is evident from the previous studies on *rpoN*-defective mutants that *rpoN* is crucial for regulating bacterial responses to resist a plethora of environmental stresses like oxidative, pH, temperature, and osmotic stress.

Therefore, the increased expression of HK (Dde_3717) and *rpoN* (Dde_3097) in elevated Cu concentrations can be due to their ability to sense Cu ions, facilitating biofilm formation and Cu resistance mechanisms.

In addition, SRB hydrogenases and cytochromes are essential components of the electron transport chain, which is crucial to supplying energy for bacterial survival and sulfur metabolism [43,70]. Our study observed comparable patterns in which the genes responsible for hydrogenase (Figure 10d) and cytochrome production (Figure 10a) were also considerably upregulated in biofilms formed in the presence of Cu. Cytochrome c3 (*cyt3*, Dde_3756) is a critical component of the ET chain, taking part in the energy metabolism of SRB. Cytochrome c3 was most upregulated in 30 µM-biofilm (log2FC = 1.772). Consequently, *cyt3* transfers electrons from cytoplasmic hydrogenases to periplasmic dissimilatory sulfate reductase during sulfate reduction. This biological ET process allows the bacteria to generate energy to support cell growth through anaerobic respiration. In addition, lactate dehydrogenases (*ldh*, Dde_3604) had the highest expression level (log2FC = 2.642) in 15 µM-biofilm samples. *Ldh* allow *Desulfovibrio* sp. to maintain energy balance and metabolic flexibility, enabling the bacteria to adapt to the changing nutrient availability by utilizing lactate as a carbon source under anoxic conditions [71,72]. The potential explanation for the increased gene expression of lactate dehydrogenases (*ldh*) and cytochrome c3 (*cyt*3) in elevated Cu concentrations can be attributed to the coordinated response of energy production and metal detoxification mechanisms, as previously reported [24,73]. Additionally, the upregulation of *ldh* and *cyt*3 can be considered an adaptive mechanism to ROS produced by the Cu ions. The bacteria can overcome this stress by increasing the ET process and enhancing its lactate utilization capacity to provide an alternative energy production pathway to meet the cell’s energy demands [24,74,75,76,77]. Other genes possibly associated with overcoming Cu stress include genes coding for NiFeSe and NiFe hydrogenases. An increase in the expression levels of *NiFe* (Dde_0082, >3-fold) and *NiFeSe* (Dde_2135, >6-fold) was observed in stress-induced biofilms compared to the controls. *NiFeSe* was found to be most upregulated in 30 µM-biofilms (>6-fold) compared to other experimental conditions. Our findings are similar to a previous study done on *D.vulgaris* under oxidative stress, suggesting the involvement of *NiFe* in stress responses [78].

It is important to note that OA G20 biofilms produced in the presence of Cu ions had a significant decrease in the expression of genes *ftsZ* (Dde_1047), *ftsA* (Dde_1046), and *ftsQ* (Dde_1045) (Figure 10b). A previous study has shown similar findings, where under Cu ion stress, the expression of the *ftsZ* gene is downregulated in planktonic cells of OA G20. *FtsZ* is a protein that plays a role in cell division, and its decreased expression can cause a delay in cell division, leading to an increase in cell size [22,79]. In our study, we also observed a similar trend in which scanning electron microscope analysis showed an increase in the size of cells when the biofilm was cultivated in a medium supplemented with Cu. Our findings can also be corroborated by a study done on *P. aeruginosa* biofilms when grown under anaerobic conditions (used as a stressor). The researchers observed that the cells became highly elongated in anaerobic biofilms. Moreover, a 10-fold decrease in the expression level of *ftsA* and *ftsZ* was observed in anaerobic biofilms compared to aerobic conditions. This change in gene expression is thought to be a survival strategy employed by the bacteria to cope with stressful conditions by modifying their cell division mechanisms [80].

Mutation studies in bacteria have revealed that the flagellum is important for bacterial biofilm formation and surface attachment. Previous studies on flagellar defective mutants of *E.coli* and *Pseudomonas putida* showed decreases in biofilm biomass and delayed cell attachment. Moreover, *fli* codes for proteins involved in the biosynthesis of the flagellar hook and basal body [81,82].In our current experiment, *fliF* (Dde_0353) was positively regulated in the test samples relative to the control. Significant upregulation compared to the control was seen in 5 µM Cu (log_2_FC = 4.763), and 30 µM Cu-biofilms (log_2_FC = 3.292), whereas some modest upregulation was observed in 15 µM Cu-biofilms (log_2_FC = 0.773) (Figure 10c). This upregulation of *fliF* (Dde_0353) in the test samples can be attributed to the bacteria’s response to Cu by augmenting their capability to move toward Cu-safe environment. The positive regulation of flagellar under stressful conditions is consistent with prior study on *E.coli*. Shuo Yang et al. reported upregulation of various genes in *E.coli* related to flagellar assembly (*flg*, *fli*), and bacterial motility when the bacteria was exposed to Rhamnolipids, a known biofilm inhibitor [83].

Upregulation (log_2_FC = 5.046) of the gene *sodB* (superoxide dismutase, Dde_0082) in the case of biofilm (30 µM Cu) was observed (Figure 10a). Reports have described the mechanisms by which Cu damages microbial cells, including Cu-precipitation and hydroxyl radical production that causes oxidative damage to macromolecules, such as proteins, lipids, and DNA, producing ROS and oxidative stress on cells. Higher concentrations of Cu can result in an overproduction of ROS, reducing sulfate utilization in bacteria. Cu also damages the cell membrane by causing oxidative stress on lipids, proteins, nucleic acids, and deactivating enzymes. Genome sequencing data indicates that *D. vulgaris* has intricate and unique mechanisms for protecting against oxidative stress, including the common ROS detoxification system found in microbes like *sodB* [16,22,84].

Bacterial EPS are produced by cells under stress and contribute to the extracellular matrix in biofilm development. The complex collective behavior of bacterial colonies involves a variety of mechanisms, such as gene regulation, EPS production, chemotaxis, motility, adhesion, and communication between cells [85,86]. Our results show that genes involved in polysaccharide biosynthesis (*poI*, Dde_3253) were positively regulated in Cu-induced biofilms (Figure 10c). Biofilms grown in 30 µM Cu were highly upregulated, with log2FC = 1.772 compared to 15 µM and 5 µM with log2FC = 0.615 and log2FC = 0.35266, respectively. *Marinobacter* and *Enterobacter cloaceae* possess the ability to bind with different metal ions. For example, EPS from *Marinobacter* displayed better adsorption of Cu ions than lead ions. In contrast, EPS from *Enterobacter cloaceae* was found to decrease the initial amounts of cadmium and Cu ions by significant levels. These bacteria synthesize EPS when exposed to metal stress, which have anionic functional groups in their structure that allow them to bind with metal ions [52,87].

## 4. Conclusions

OA G20 adopts several mechanisms to protect the cells from heavy metal stress when exposed to Cu stress. Key strategies adopted by bacteria to resist metal ion stress are promoting biofilm formation, EPS production, and the regulation of gene expression. Metal exposure not only leads to phenotypic changes in OA G20 but can also alter the structure of the biofilm matrix. Stress-induced biofilms can change their EPS composition to enhance the biofilm’s ability to trap metal ions. Additionally, gene expression analysis revealed that resistance to Cu in OA G20 is a complex phenomenon which cannot be explained by just one mechanism. In this study, we evaluated the influence of bioavailable Cu on the biofilm formation of OA G20 on a glass surface. The outcomes observed in this study include: (a) OA G20 biofilms showed increased resistance to Cu ions in comparison to planktonic cells; (b) SEM and CV studies revealed enhanced biofilm formation in the presence of 30 µM Cu relative to controls; (c) More EPS production was observed using protein and carbohydrate analysis on Cu-induced (5, 15, and 30 µM) biofilms compared to controls; d) Cell attachment was observed at 100 µM Cu, suggesting that higher Cu concentration is required to inhibit biofilm formation in OA G20; (e) Genes involved in sulfur metabolism, polysaccharide biosynthesis, electron transport, and stress response were positively regulated in Cu-induced biofilms; (f) *ftsZ*, *ftsA*, *ftsQ*- genes involved in cell division were downregulated compared to the control, suggesting the involvement of these genes in the cell elongation observed in Cu-induced biofilms. Our results provide a baseline for future studies of the mechanisms of Cu-induced biofilms of OA G20. Nonetheless, biofilm formation under metal stress requires further investigation, as biofilms merely do not act as a physical barrier to the diffusion of toxic compounds. Instead, the cells within these biofilms utilize specific mechanisms to counteract metal stress. Proteomic analysis of the biofilms is necessary to validate the mechanisms employed by bacteria to survive Cu toxicity.

## Data Availability

The original contributions presented in the study are included in the article/Supplementary Materials, further inquiries can be directed to the corresponding author.

## Supplementary Materials

The following supporting information can be downloaded at: https://www.mdpi.com/article/10.3390/microorganisms12091747/s1, Table S1: Media composition; Table S2: Forward and Reverse primer sequences for RT-qPCR; Figure S1: Images of CV staining for OA G20 biofilms grown in 96-well plates in variable Cu concentrations (5,15, and 30 µM) on days 1(1a), 3(1b), 5(1c), and 7(1d); Figure S2: Images of CV staining for OA G20 biofilms grown on GC in 12-well plates in variable Cu concentrations (5,15, and 30 µM) on days 1(2a), 3(2b), and 5(2c); Figure S3: (a) Growth curve of OA G20 in lactate-C medium supplemented with 60, 80, and 100 µM Cu; (b) Influence on OA G20 growth as a function of total protein (OD660 nm) evaluated at variable Cu ion concentrations. Figure S4: Images of CV staining for OA G20 biofilms grown in 96-well plates in variable Cu concentrations (60,80, and 100 µM) on days 1(4a), 3(4b), 5(4c), and 7(4d).
