# Supplementary material for: Influence of Copper on Oleidesulfovibrio alaskensis G20 Biofilm Formation
Source: Microorganisms. 2024 Aug 23;12(9):1747. doi: 10.3390/microorganisms12091747 (PMC11434458; doi:10.3390/microorganisms12091747)
Supplement: Supplementary file 1 [file microorganisms-12-01747-s001.zip › Supplementary tables/Supplementary Table S2.pdf]

Table S2: Forward and Reverse primer sequences for RT-qPCR

Gene names	Gene Id	Protein Names	Primer name	Primer sequence (5'→ 3')
		**Sulfur metabolism**		
dsrA	Dde_0526	Sulfite reductase, dissimilatory-type alpha subunit	Forward<br>Reverse	ACCAGCCTGCAGGTAAGTTC<br>CGTGGGTCAGTTCCCAGAAA
dsrB	Dde_0527	sulfite reductase, dissimilatory-type beta subunit	Forward<br>Reverse	GGCTGAATCCGGCGAAAAAG<br>CGCAGAGCTTTCATGGCTTC
sat	Dde_2265	sulfate adenylyltransferase	Forward<br>Reverse	CGCTGTTGAAGTGTGTGACG<br>CGTAGTTCTGGCGGAAGGTT
aprA	Dde_1109	adenylylsulfate reductase, subunit A	Forward<br>Reverse	GCTTACAACCAGGAACCCGA<br>TGCCGTTACGGAACTTGACA
		**Electron Transport**		
NiFeSe*	Dde_2135	Periplasmic (NiFeSe) hydrogenase, large subunit	Forward<br>Reverse	ACCCCTTTAACCTTGTGGGC<br>TACAGACACCACCCTTTGCG
NiFe*	Dde_0082	Periplasmic (NiFe) hydrogenase large subunit	Forward<br>Reverse	TGTTCCCGCTCAATGACGAA<br>TTCGGGGTCAGGTATGGTCT
ldh	Dde_3604	D-lactate dehydrogenase	Forward<br>Reverse	AACAGGTCAGCCGCATAGTC<br>GCTCTCCGGTAATCACTCCG
cyt3	Dde_3756	Cytochrome-c3 hydrogenase	Forward<br>Reverse	TTTCGGATTCACCCGCATCA<br>ACAAATCCAGCCACCGTCAT
		**Cell division**		
FtsA	Dde_1046	Cell division protein FtsA	Forward<br>Reverse	GCGACGACGAGGGAATAGAG<br>GATCGACCAGCGACAGGATT
FtsQ	Dde_1045	Polypeptide-transport-associated domain protein FtsQ-type	Forward<br>Reverse	CAGATAGACTCCGAAGCCGC<br>TGCCTGCTGTCAGGTGTATG
FtsZ	Dde_1047	Cell division protein FtsZ	Forward<br>Reverse	TTTATCACCGCCAACACCGA<br>CTTTCAAGGGCTGCCTGTCT
		**Flagellar biosynthesis**		
fliF	Dde_0353	Flagellar M-ring protein	Forward<br>Reverse	ATGTCCATTCGCCCGTTTCT<br>CGGAAGACTCCAACCGTGAA
		**Two-component**		
rpoN	Dde_3097	Sigma-factor rpoN	Forward<br>Reverse	CACTGAAATCCGGCAAAGCC<br>GAATCTTCCGGCGCTTACCT
HK*	Dde_3717	sensor signal transduction histidine kinase	Forward<br>Reverse	TTTAAAACAGCCTGGCCCGA<br>CAGCGCAAATTCCAGCACAT
		**Stress Response**		
sodB*	Dde_0882	Superoxide dismutase	Forward<br>Reverse	AACCGCCAATGCCGAAAATC<br>AGATGGTCAAGAAACGCGGA
		**Polysaccharide synthesis**		
poI*	Dde_3253	Capsule polysaccharide biosynthesis protein	Forward<br>Reverse	TCGCCGTGTTCGTGGTATTT<br>AAGCAGAAAGTCCATGCCGA

**Housekeeping gene**				
recA	Dde_2373	Recombinase A	Forward	GATCGCCGAATGCCAGAAAC
			Reverse	TCGGCGATATCAAGTGCCTG

*These gene name abbreviations are not annotated in databases and the abbreviations are only used to enhance readability.
