# Supplementary figures and images for: Influence of Copper on Oleidesulfovibrio alaskensis G20 Biofilm Formation
Source: Microorganisms. 2024 Aug 23;12(9):1747. doi: 10.3390/microorganisms12091747 (PMC11434458; doi:10.3390/microorganisms12091747)

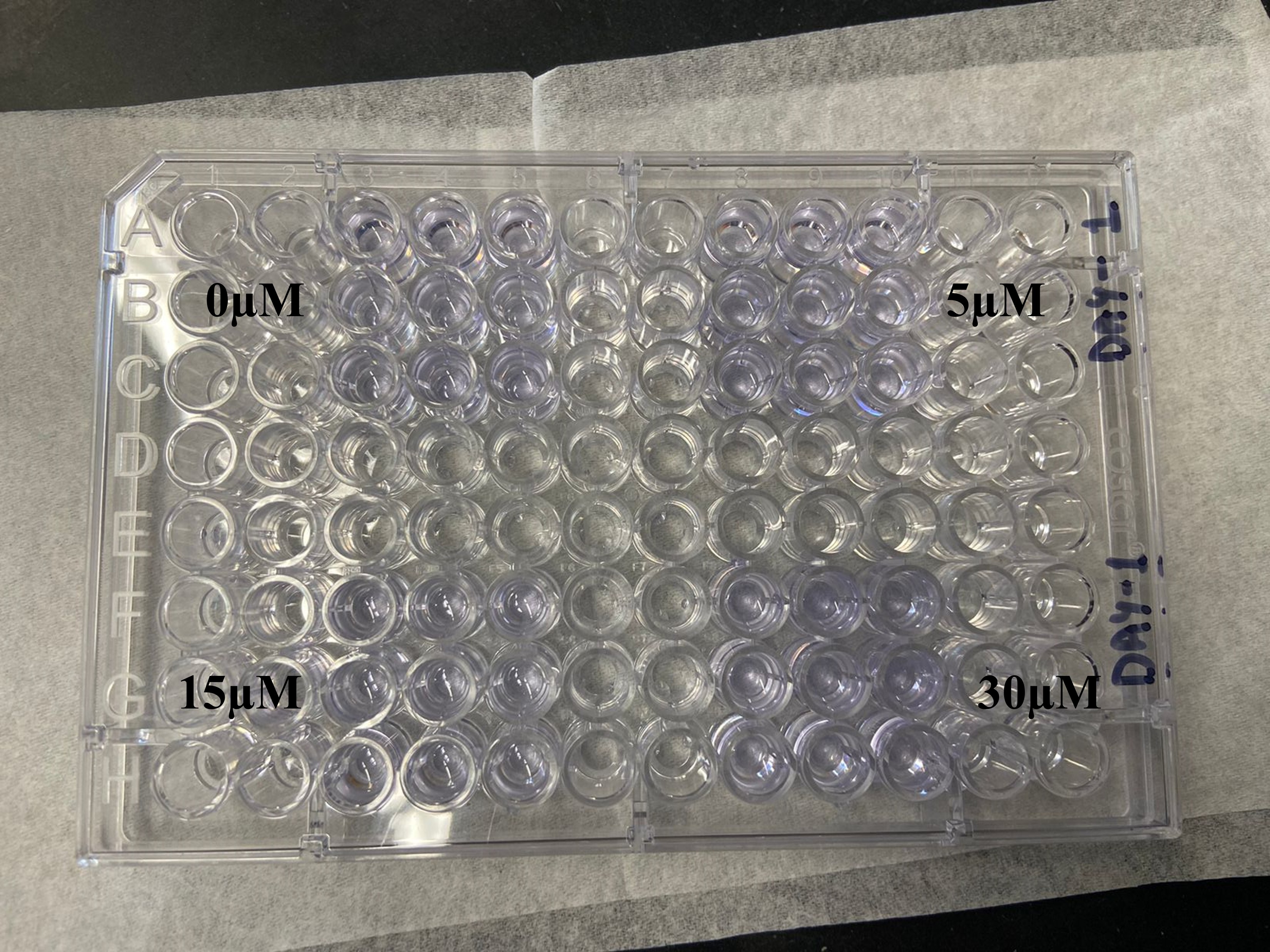

Supplement: Supplementary file 1 [file microorganisms-12-01747-s001.zip › Supplementary Figures/Supplementary Figure 1a.jpg]

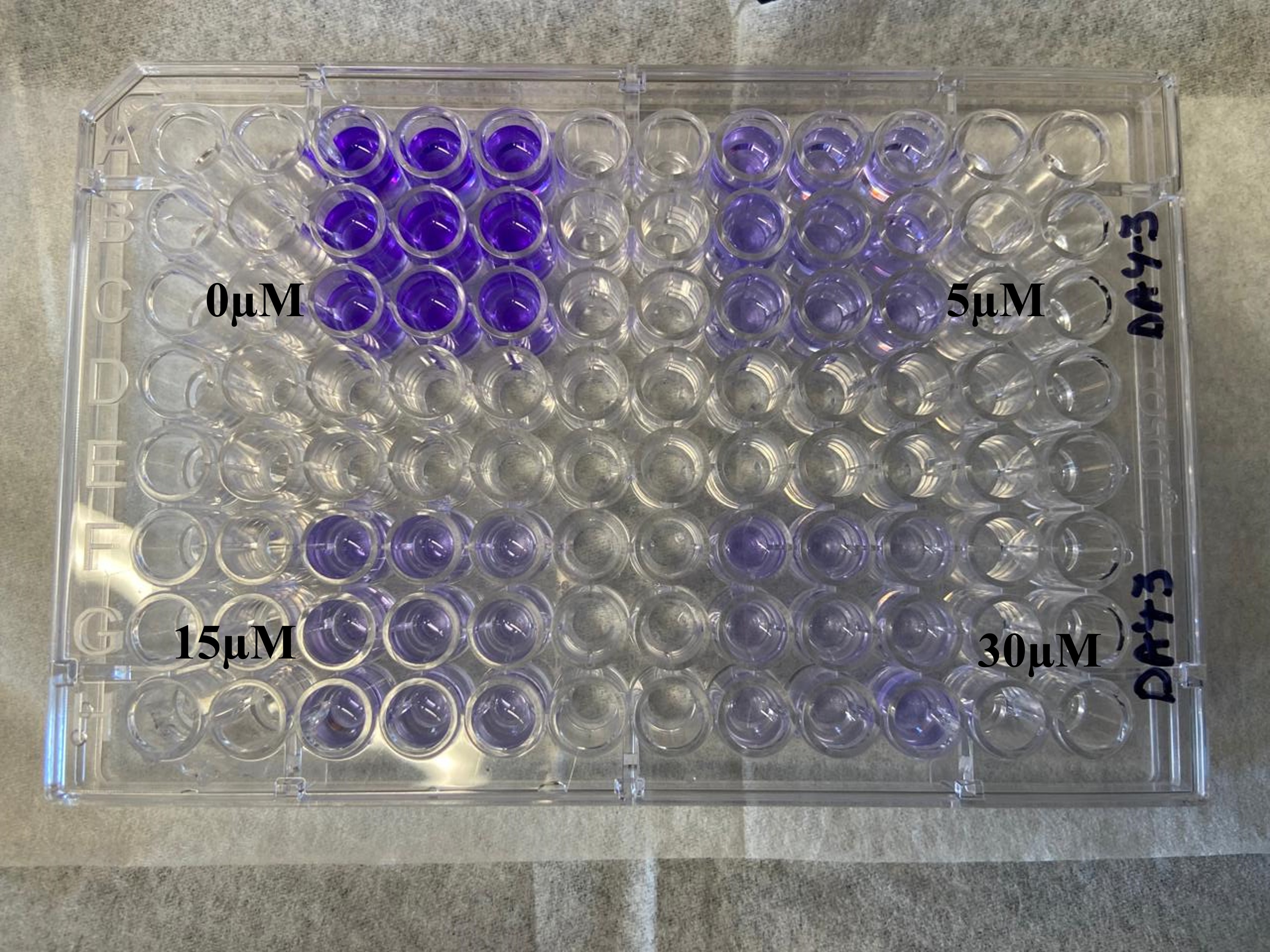

Supplement: Supplementary file 1 [file microorganisms-12-01747-s001.zip › Supplementary Figures/Supplementary Figure 1b.jpg]

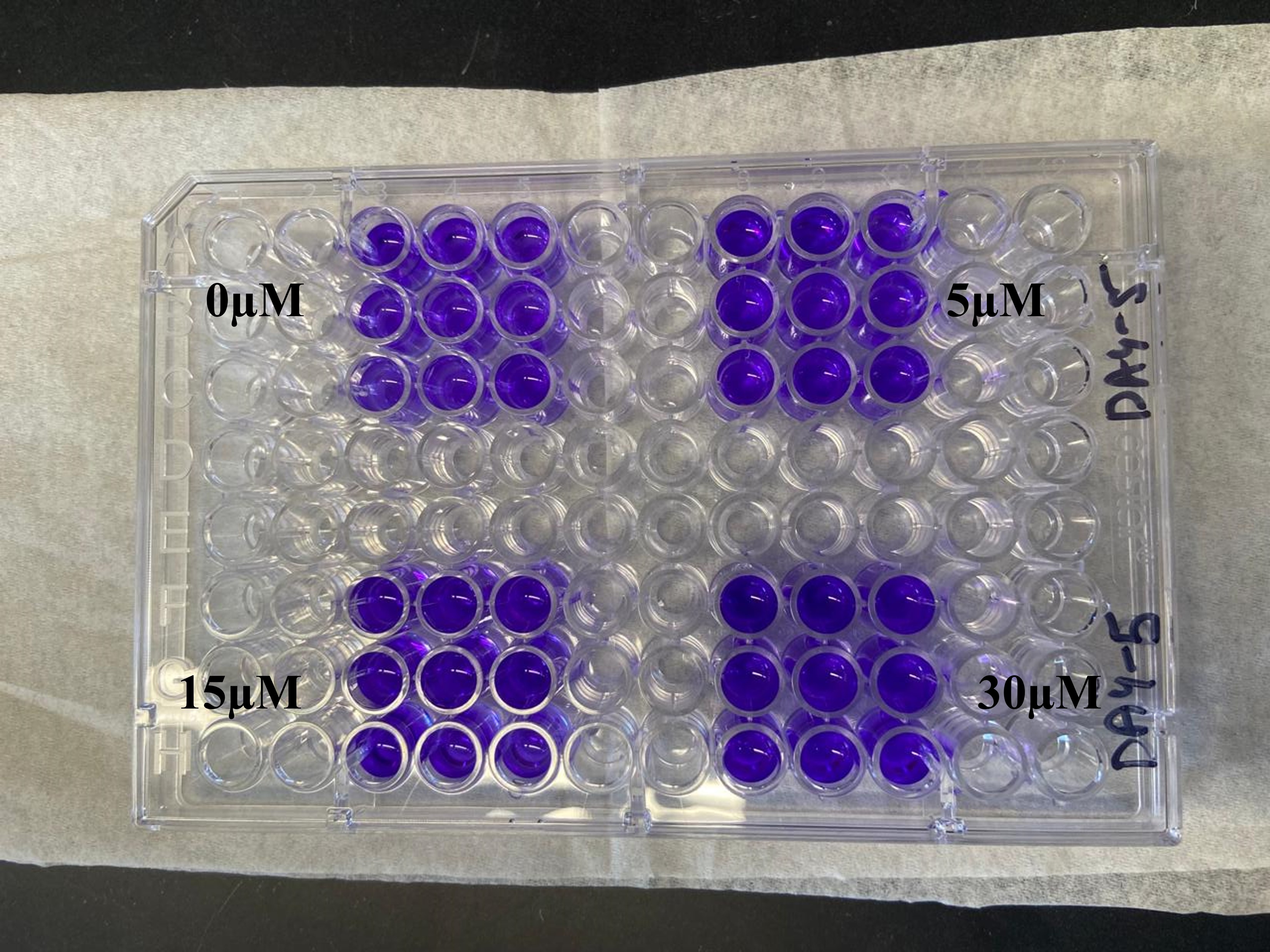

Supplement: Supplementary file 1 [file microorganisms-12-01747-s001.zip › Supplementary Figures/Supplementary Figure 1c.jpg]

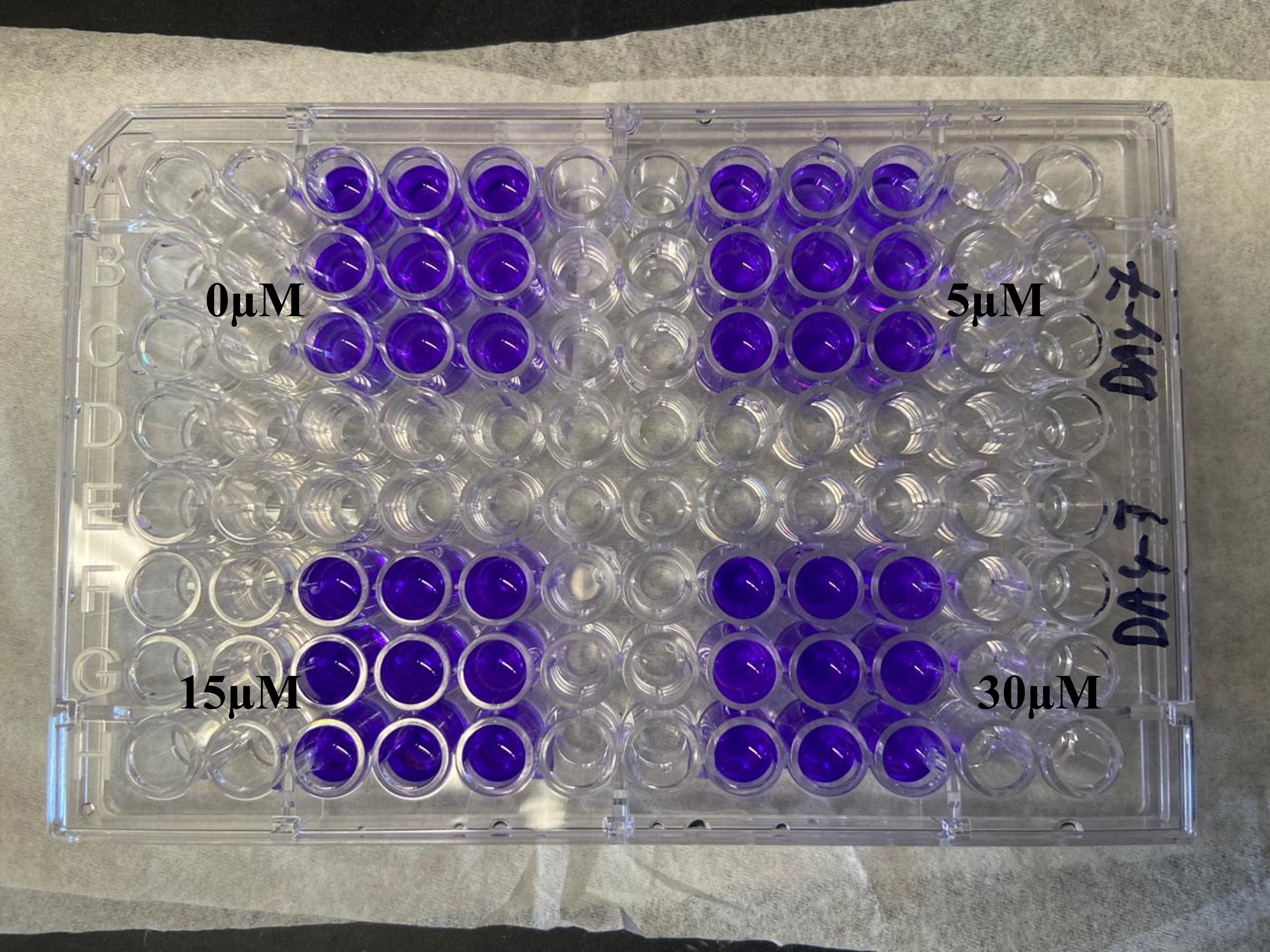

Supplement: Supplementary file 1 [file microorganisms-12-01747-s001.zip › Supplementary Figures/Supplementary Figure 1d.jpg]

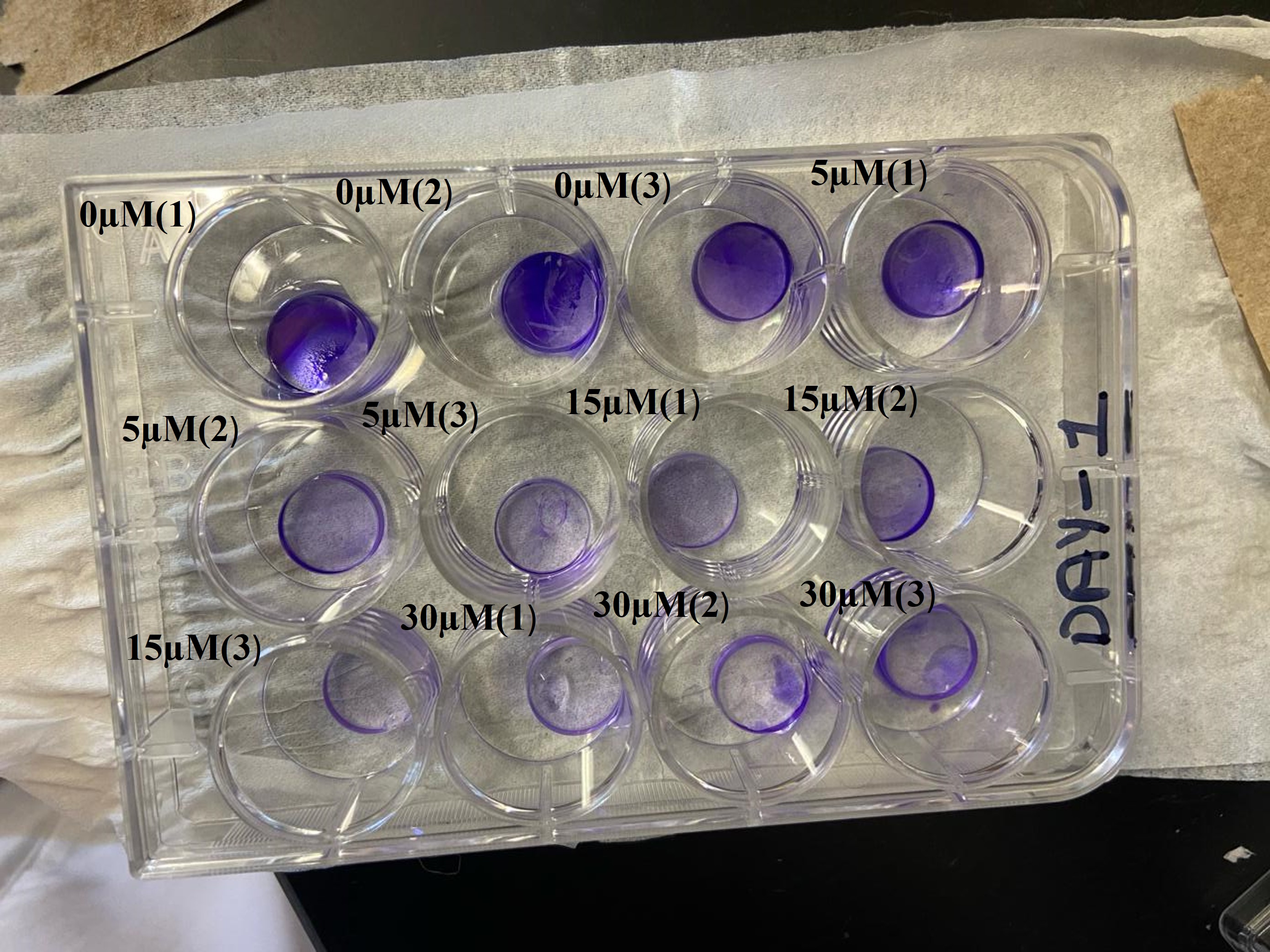

Supplement: Supplementary file 1 [file microorganisms-12-01747-s001.zip › Supplementary Figures/Supplementary Figure 2a.jpg]

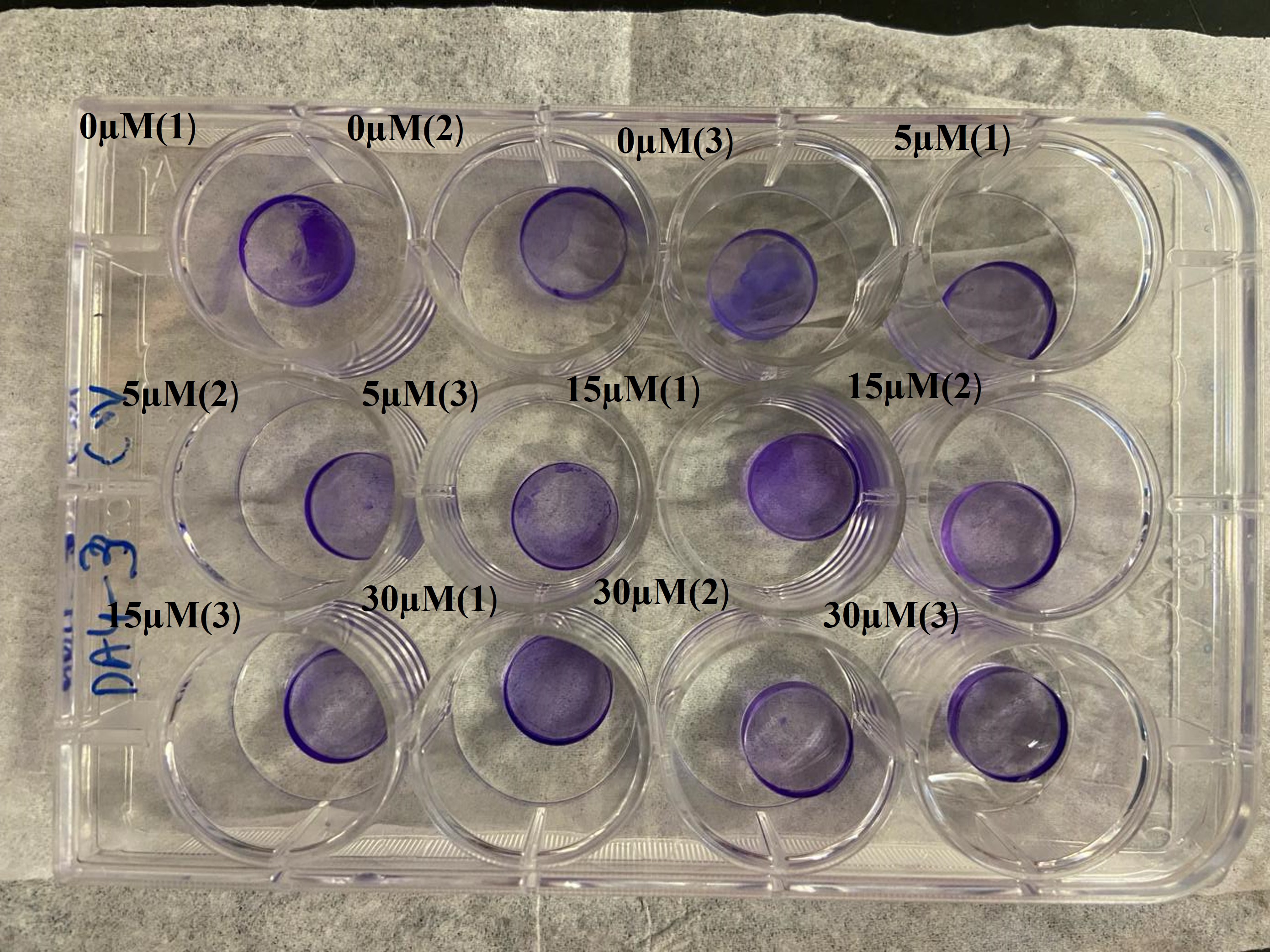

Supplement: Supplementary file 1 [file microorganisms-12-01747-s001.zip › Supplementary Figures/Supplementary Figure 2b.jpg]

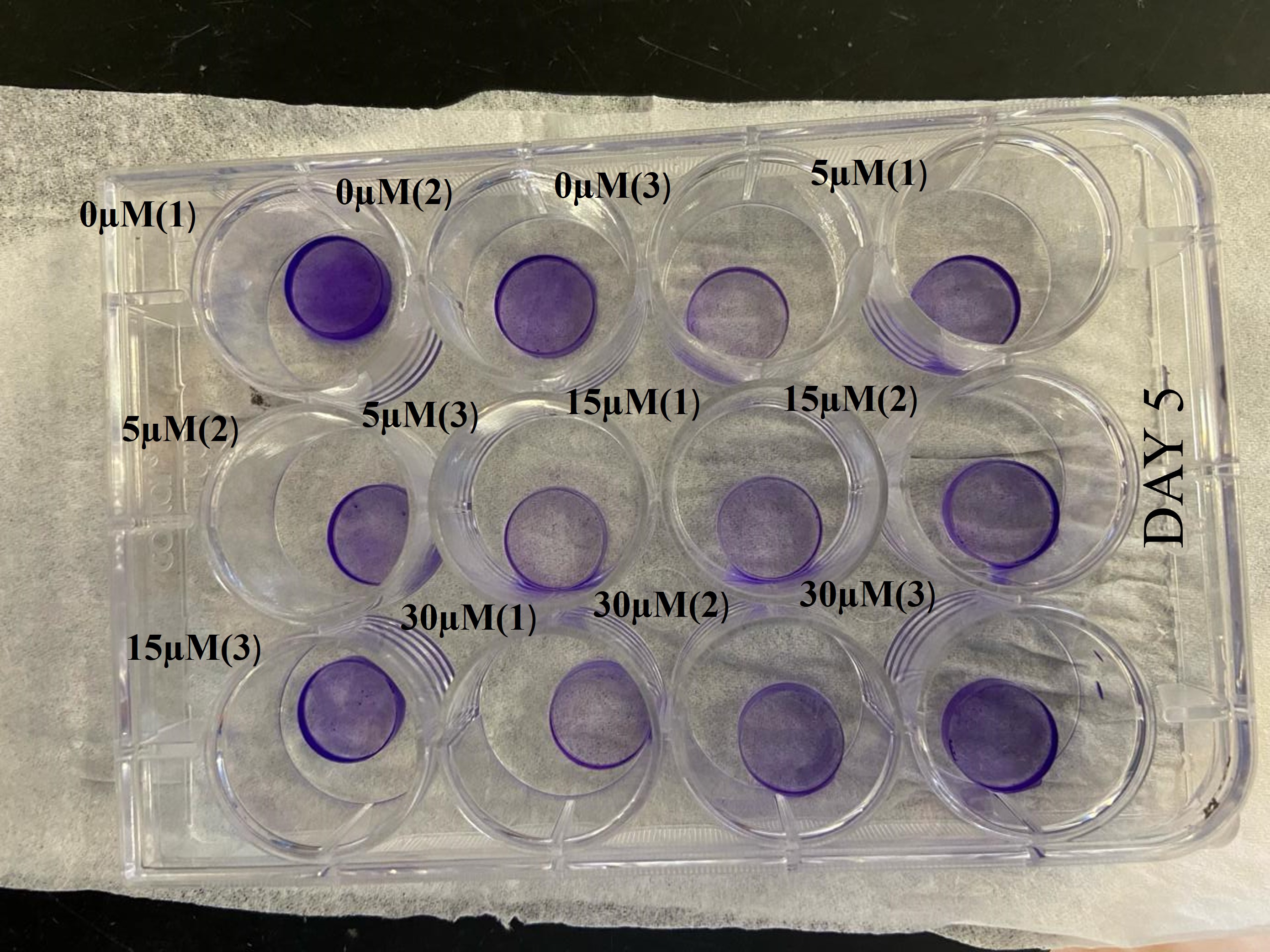

Supplement: Supplementary file 1 [file microorganisms-12-01747-s001.zip › Supplementary Figures/Supplementary Figure 2c.jpg]

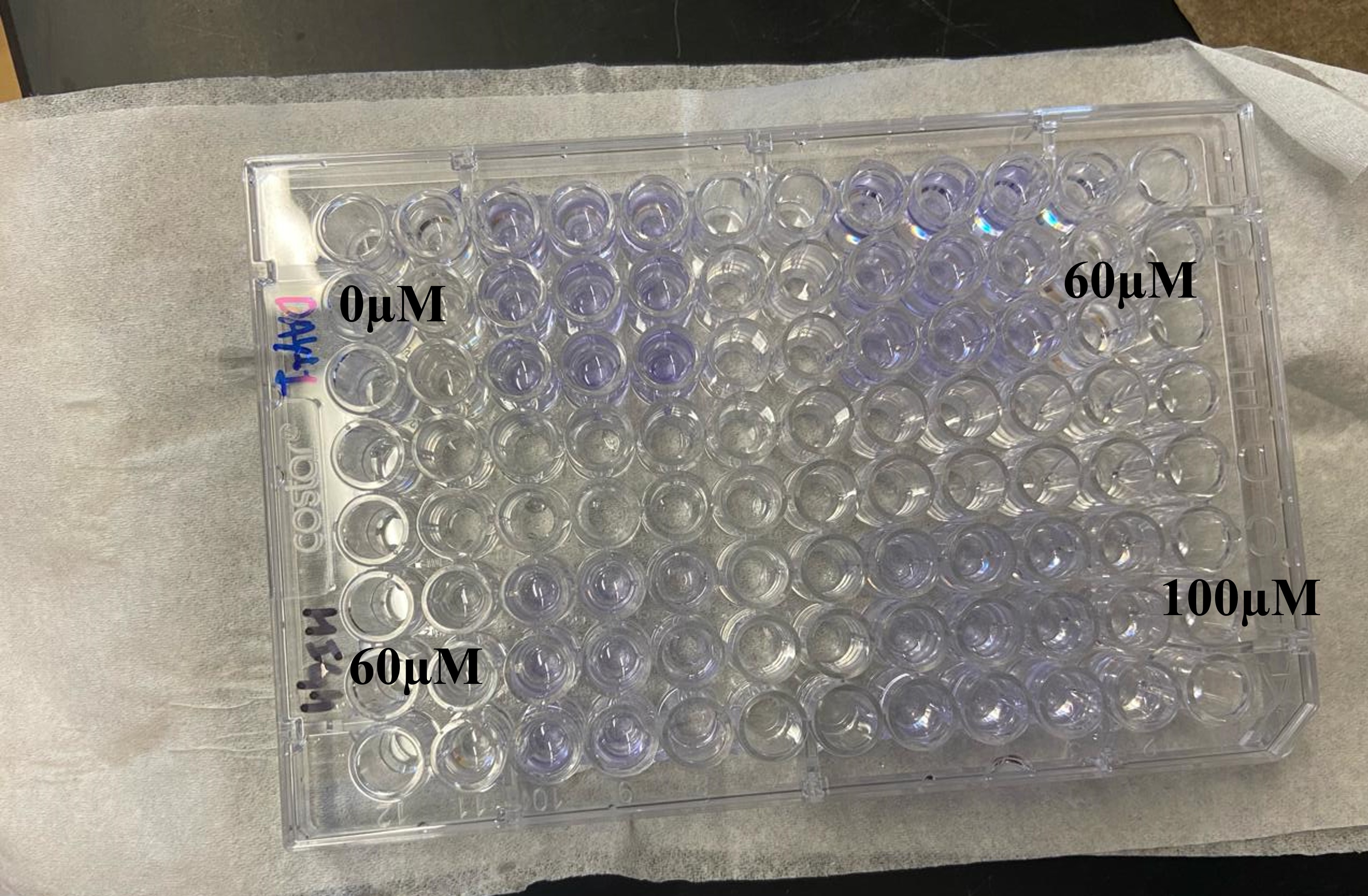

Supplement: Supplementary file 1 [file microorganisms-12-01747-s001.zip › Supplementary Figures/Supplementary Figure 4a.jpg]

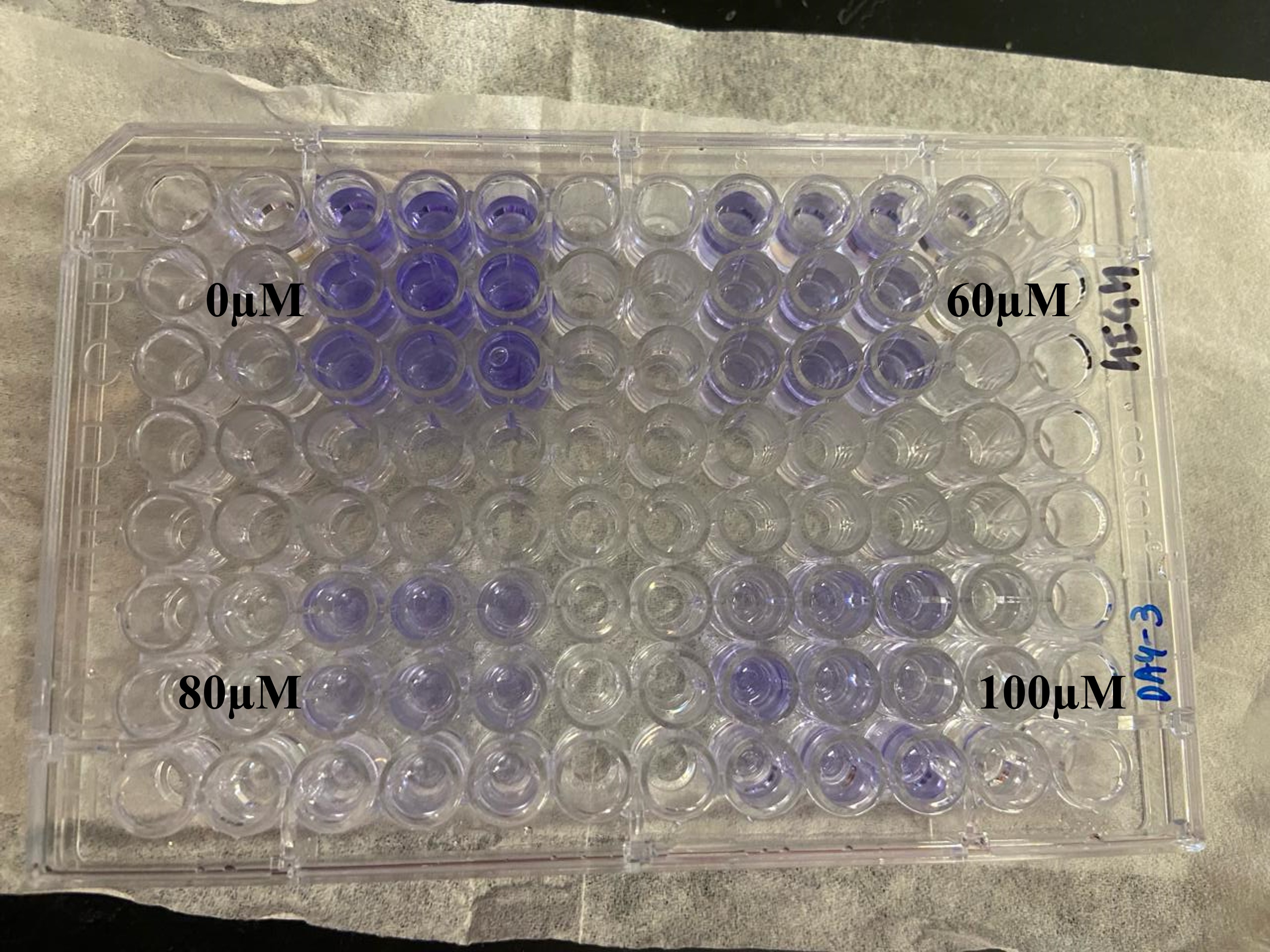

Supplement: Supplementary file 1 [file microorganisms-12-01747-s001.zip › Supplementary Figures/Supplementary Figure 4b.jpg]

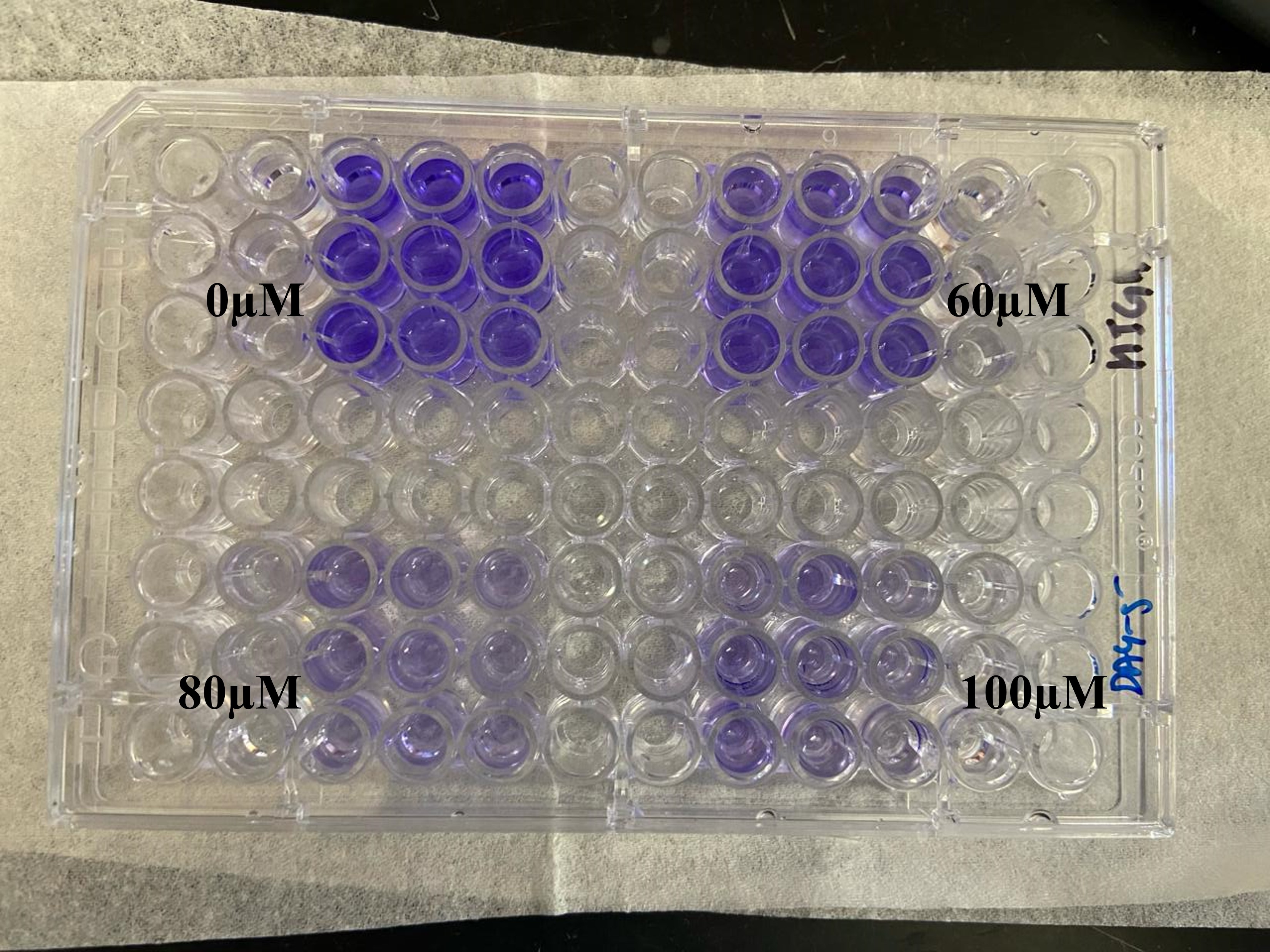

Supplement: Supplementary file 1 [file microorganisms-12-01747-s001.zip › Supplementary Figures/Supplementary Figure 4c.jpg]

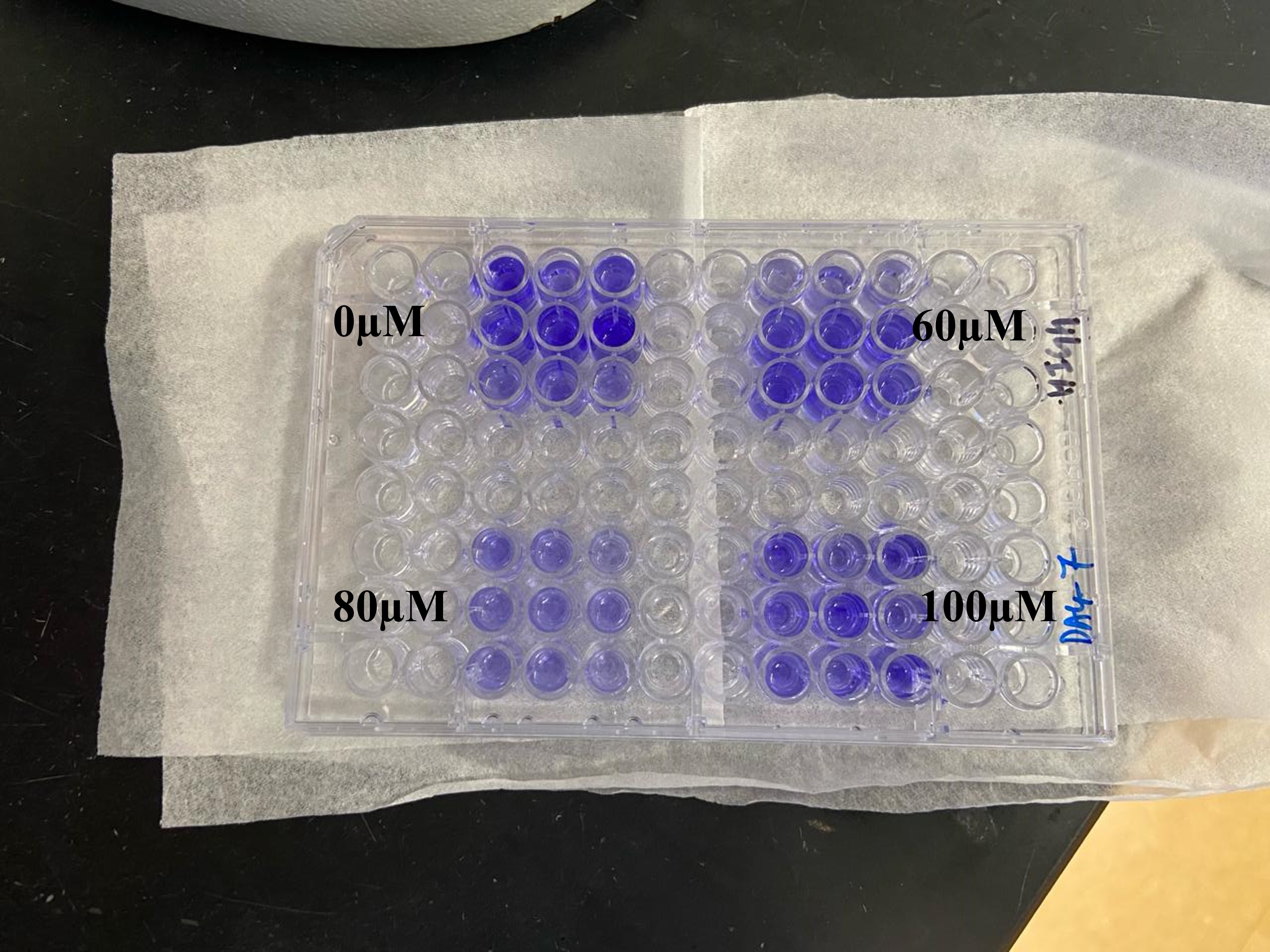

Supplement: Supplementary file 1 [file microorganisms-12-01747-s001.zip › Supplementary Figures/Supplementary Figure 4d.jpg]
